# The role of snare proteins in cortical development

**DOI:** 10.1002/dneu.22892

**Published:** 2022-07-05

**Authors:** Auguste Vadisiute, Elise Meijer, Florina Szabó, Anna Hoerder‐Suabedissen, Eri Kawashita, Shuichi Hayashi, Zoltán Molnár

**Affiliations:** ^1^ Department of Physiology, Anatomy and Genetics, Sherrington Building University of Oxford Oxford UK; ^2^ Department of Pathological Biochemistry Kyoto Pharmaceutical University Yamashina‐ku Kyoto Japan; ^3^ Department of Anatomy Kawasaki Medical School Kurashiki Okayama Japan

**Keywords:** Munc13, Munc18, myelination, neuronal activity, Snap25, SNARE complex, synapse, synaptic vesicle release

## Abstract

Neural communication in the adult nervous system is mediated primarily through chemical synapses, where action potentials elicit Ca^2+^ signals, which trigger vesicular fusion and neurotransmitter release in the presynaptic compartment. At early stages of development, the brain is shaped by communication via trophic factors and other extracellular signaling, and by contact‐mediated cell–cell interactions including chemical synapses. The patterns of early neuronal impulses and spontaneous and regulated neurotransmitter release guide the precise topography of axonal projections and contribute to determining cell survival. The study of the role of specific proteins of the synaptic vesicle release machinery in the establishment, plasticity, and maintenance of neuronal connections during development has only recently become possible, with the advent of mouse models where various members of the N‐ethylmaleimide‐sensitive factor attachment protein receptor (SNARE) complex have been genetically manipulated. We provide an overview of these models, focusing on the role of regulated vesicular release and/or cellular excitability in synaptic assembly, development and maintenance of cortical circuits, cell survival, circuit level excitation–inhibition balance, myelination, refinement, and plasticity of key axonal projections from the cerebral cortex. These models are important for understanding various developmental and psychiatric conditions, and neurodegenerative diseases.

## 1 INTRODUCTION

Communication between neurons is fundamental to the development and function of the nervous system. The earliest communication is through gap junctions and electrical synapses between newly born, still migrating neurons.

**FIGURE 1:**
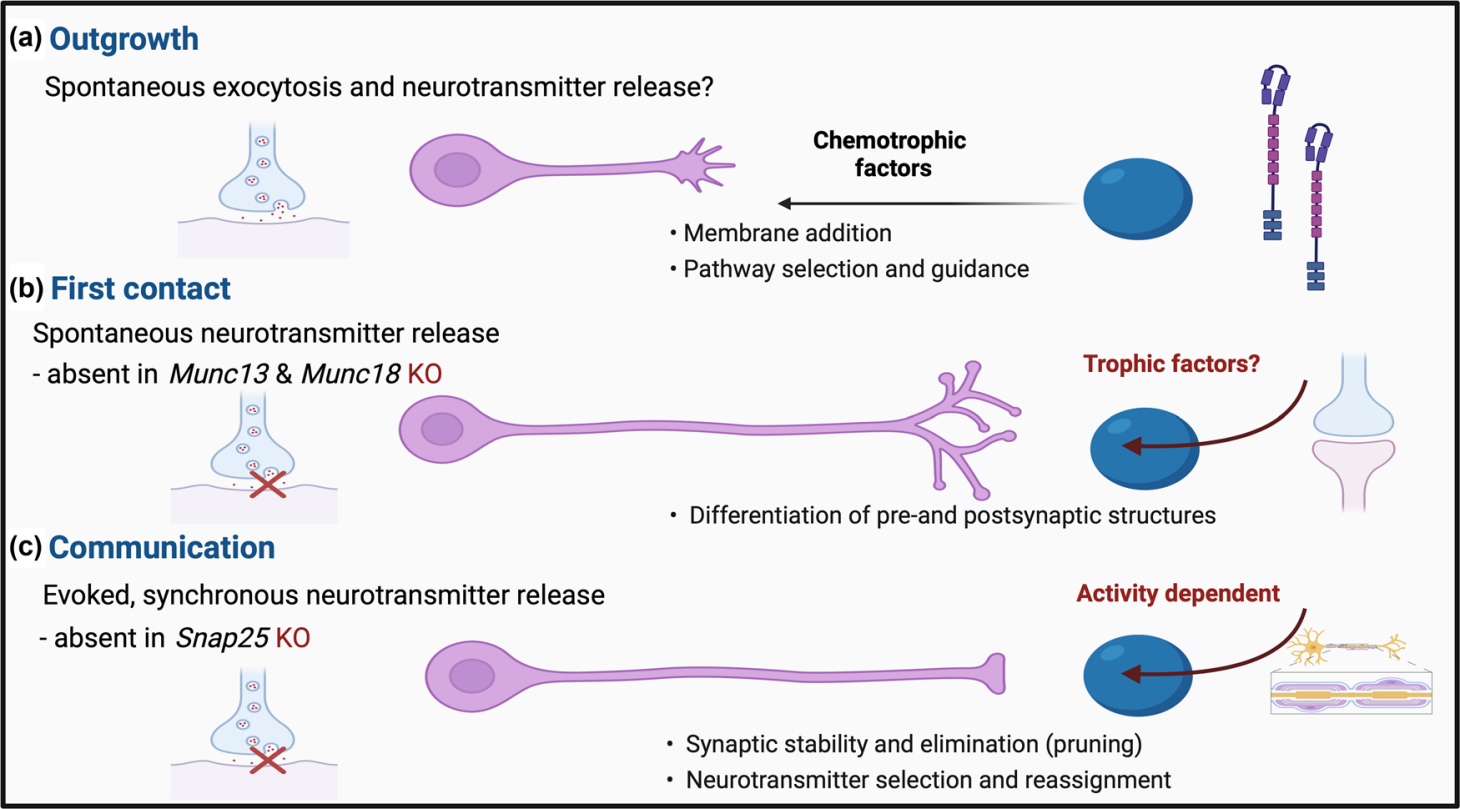
When does spontaneous and regulated synaptic vesicle release play a part in neuronal development? We are interested in the stages when spontaneous and regulated synaptic vesicular release is involved in neuronal development. (a) Does axon outgrowth only rely on spontaneous and not regulated neurotransmitter release? Is membrane addition, axon outgrowth, pathway selection and guidance dependent on regulated synaptic vesicular release? (b) First contact to establish synapses is present even in the absence of neurotransmitter release. (c) Neuronal communication is dependent on evoked, synchronous neurotransmitter release. Created with BioRender.com

Next, neurotransmitters start being released by spontaneous fusion of neurotransmitter vesicles with the cell membrane (Andreae et al., 2012; Dagani & D'Angelo, 1992; Fatt & Katz, 1952; Katz & Miledi, 1963; Mozhayeva et al., 2002), and paracrine release where neurotransmitter escapes the neuron independent of the vesicular machinery (Demarque et al., 2002) (Figure 1). Neurotransmitter release has been demonstrated in growing axons before target contact and synapse formation, suggesting that spontaneous neurotransmitter release could play a role in pathway guidance and target selection (Andreae & Burrone, 2018, 2015; Girod et al., 1995; Hume et al., 1983; Taylor et al., 1990; Verderio, Coco, Pravettoni et al., 1999; Young & Poo, 1983).

With the maturation of sensory organs and their connections to the nervous system, neuronal activity will be modulated by sensory‐driven activity patterns. At this stage, neural communication is increasingly mediated through chemical synapses, where neurotransmitter release is evoked from docked vesicles by Ca^2+^ signals triggered by presynaptic action potentials (Matthews, 1996). The spontaneous and sensory‐driven activity patterns can influence other neurons in multiple ways, such as by regulating production and release of growth factors, maintenance of gap junctions, maintenance or elimination of transient cells, remodeling of somatodendritic morphology, formation and maintenance of synapses, and myelination of axons (Katz & Shatz, 1996; Maffei, 2002; West & Greenberg, 2011). The regulated neurotransmitter release elicited by early patterns of activity may further guide the precise topography of neuronal projections and their circuit assembly (Katz & Shatz, 1996; Matthews, 1996), and overall levels of activity of the circuits, which will subsequently determine cell survival, including that of interneurons (Wong et al., 2018).

There is increasing evidence suggesting that transient alterations in neuronal activity during restricted postnatal periods can lead to persistent changes in functional connectivity and even cell distribution and therefore might underlie the manifestation of pathological conditions. Fundamental knowledge on early steps of activity dependent synapse formation and maintenance does not only have major general biological implications but is also key for better understanding of the pathogenesis of neurological and neuropsychiatric disorders.

Early studies on the role of synaptic communication in the developing brain interfered with the activity of entire neuronal populations, predominantly by infusion of the sodium channel blocker tetrodotoxin (TTX) during development in vivo (Harris, 1984; Katz & Shatz, 1996; Stryker & Harris, 1986). TTX binds to voltage‐gated sodium channels in nerve cell membranes and inhibits firing of action potentials, therefore allowing researchers to investigate the necessity of action potentials for a biological process of interest. However, infusion of TTX is neither selective to synaptic transmission nor able to selectively target neuronal populations during specific phases of development. Moreover, such interventions typically have effects on various other biological processes that can confound results drastically and yield contradictory results in vivo and in vitro (Kossel et al., 1997; McKinney et al., 1999; Soares et al., 2013).

Recent models are more selective and take advantage of specific proteins that form part of the synaptic machinery. Since Ca^2+^ regulated evoked release and spontaneous vesicular release, and constitutive nonvesicular release of neurotransmitters all make use of specific components of the synaptic machinery, eliminating individual components allows the investigation of the relative contributions of these different forms of neuronal signaling (Rizo & Südhof, 2012). These manipulations can be performed in a cell‐type specific manner, permitting the study of the role of the respective synaptic components in different brain structures independently. Moreover, by limiting removal of essential proteins to certain brain areas, embryonic lethality of some mouse models can be avoided, so that various stages of postnatal development can be studied, including myelination and synaptic maturation/maintenance.

In this review, we aim to give an overview of historic and recent studies on the role of regulated and spontaneous vesicle release in cortical development. We discuss findings from in *vitro* studies and the results of in *vivo* studies with transgenic mouse models on the role of Synaptosomal‐associated protein 25 (Snap25), Munc13 and Munc18, Syntaxin, and Synaptobrevins/vesicle‐associated membrane proteins (VAMPs) in cortical development. We will also describe the implications of the findings from mouse studies for the understanding of neurodevelopmental and neurodegenerative diseases. We conclude with outstanding questions that should be studied with newly available techniques in the years to come.

### 1.1 SNARE complex

The soluble N‐ethylmaleimide fusion protein attachment protein receptor (SNARE) complex is universally involved in membrane fusion (Han et al., 2017; Jena, 2011; Pobbati et al., 2006) synaptic vesicle trafficking (Hay, 2001; Nicholson et al., 1998), and neuronal maintenance (Barrecheguren et al., 2017; Ulloa et al., 2018; Washbourne et al., 2002). SNARE proteins are characterized by SNARE motifs, 65‐residue stretches of amino acids that tend to form coiled coils in tertiary structure. The central components of the SNARE complex are Snap25, VAMP/ synaptobrevin, and syntaxins. Other essential proteins such as Munc13 and 18, synaptophysin, and synaptotagmin interact with and regulate the formation of the SNARE complex. Snap25 is necessary for regulated synaptic vesicle release, and Munc13 and Munc18 are involved in spontaneous synaptic vesicle release. SNARE expression levels change throughout life, suggesting a differential role of these proteins in development and maturation of neuronal connections (Greenlee et al., 2001, 2002; Washbourne et al., 2002).

**FIGURE 2:**
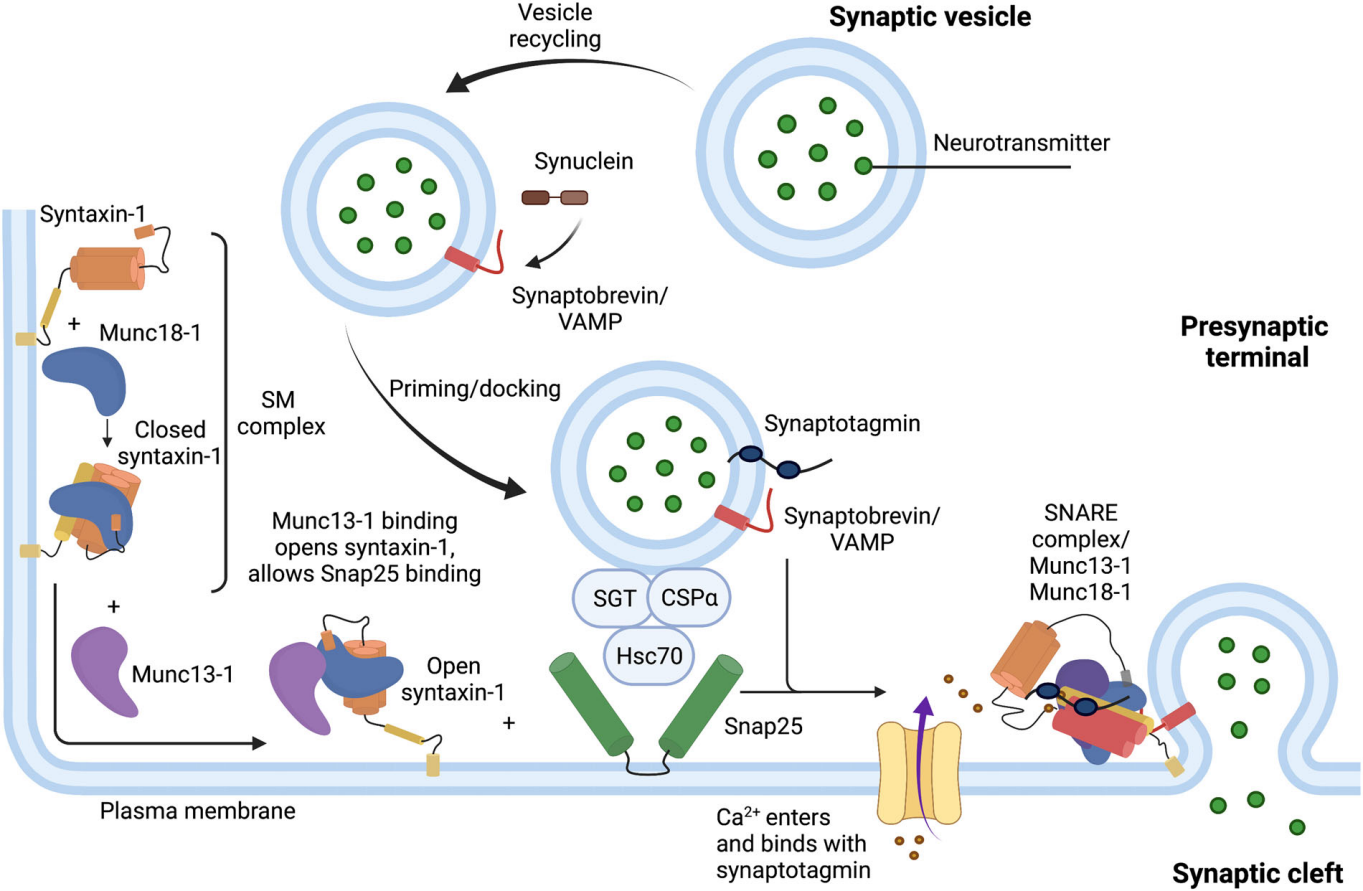
Overview of the functions of SNAREs, Munc18‐1, and some of their accomplices at the synaptic terminal modified from Rizo and Südhoff (2012). Synaptobrevin (red) is shown on synaptic vesicles as a largely unstructured protein that binds to synuclein (brown). Syntaxin‐1 [Habc domain and N‐peptide (orange), SNARE motif (yellow)] is anchored on the plasma membrane and initially forms a closed conformation that binds to Munc18‐1 (blue). Snap25 (green) is maintained in a state that is competent for SNARE‐complex assembly by interactions with Hsc70 and its cochaperones CSPα and SGT. Synaptic vesicle fusion requires assembly of the SNARE complex (bottom right), which is orchestrated in part by Munc18‐1 and the Munc13‐1 MUN domain (purple). Munc18‐1 and perhaps also the MUN domain likely cooperate with the SNAREs in inducing membrane fusion. Synuclein assists in SNARE‐complex assembly by as‐yet unidentified interactions. After fusion, the SNARE complex is disassembled by NSF and Snaps, and synaptobrevin is sorted to the synaptic vesicles. The same color coding is used in all the figures, except that Munc18‐1 is depicted with different shades of blue in Figure 2. CSP, cysteine string protein; NSF, N‐ethylmaleimide sensitive factor; SGT, small glutamine‐rich protein; SNAPs, soluble NSF adaptor proteins; SNARE, SNAP receptor; Snap25 synaptosomal associated protein of 25 kDa. Created with BioRender.com

During membrane fusion, SNARE proteins on transport vesicles and nerve terminal membranes combine into a multi‐helix trans‐SNARE complex (Rizo & Südhof, 2012; Sollner et al., 1993). The formation of the SNARE complex mediates exocytosis by facilitating close interaction of the vesicle membrane with the plasma membrane (Figure 2). Upon Ca^2+^ influx through voltage‐gated calcium channels in the presynaptic compartment, the two SNARE motifs of Snap25 interact with those of synaptobrevin in the synaptic vesicle membrane and syntaxin‐1 in the plasma membrane, respectively, to form a four‐helix bundle. As synaptobrevin and syntaxin‐1 are membrane‐anchored by transmembrane domains, and Snap25 is secured in the membrane by palmitoylated cysteine residues, this interaction leads to the approximation of synaptic vesicle with the presynaptic cell membrane, resulting in synaptic vesicle exocytosis (Hanson et al., 1997; Poirier et al., 1998; Sutton et al., 1998).

The SNARE complex is extremely stable, and for it to disassemble, hydrolysis of Adenosine 5'‐triphosphate (ATP) by N‐ethylmaleimide sensitive factor (NSF) is necessary (Sollner et al., 1993). Thus, SNARE complex assembly is assumed to provide the energy for synaptic vesicle fusion (Banerjee et al., 1996; Mayer et al., 1996). Moreover, the necessity of the formation of the SNARE complex for synaptic vesicle release introduces a point of regulation, since the N‐terminals of various SNARE proteins contain regulating sequences (Rizo & Südhof, 2012).

**FIGURE 3:**
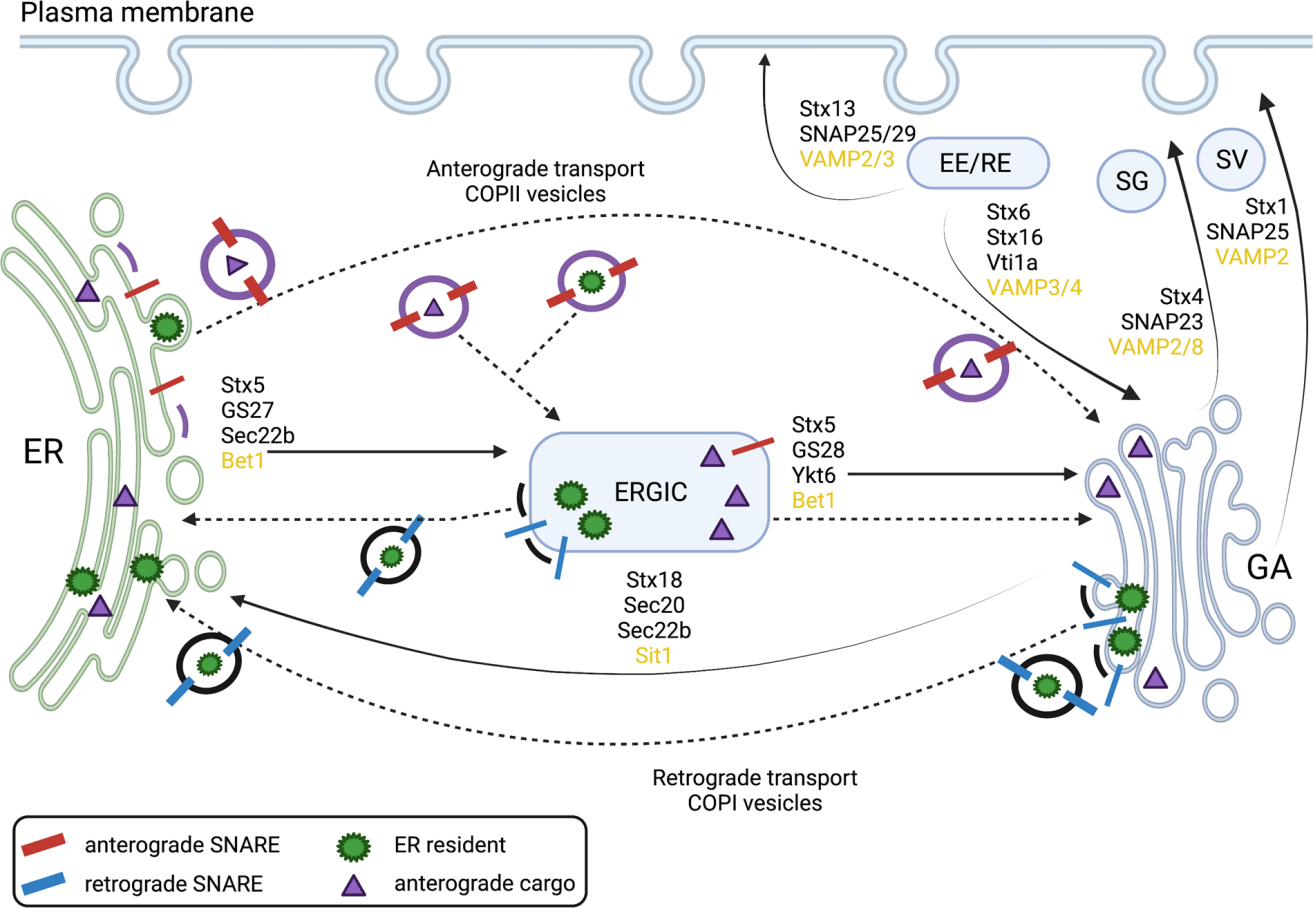
The SNARE complex proteins in vesicular transport. Summary of known SNARE complex proteins and their sites of action in various transport pathways. SNARE proteins labelled in yellow (Bet1, Sit1, VAMPs) bound to vesicle membrane, other proteins involved in vesicular transport labelled in black (Stx–syntaxin, GSs, Vit1a, SNAPs, Ykt6, Secs). EE, early endosome; ER, endoplasmic reticulum; ERGIC, ER‐Golgi intermediate compartment; GA, Golgi apparatus; SG, secretory granule; SV, synaptic vesicle; RE, recycling endosome; COPI, coat protein‐I; COPII, coat protein‐II. Sec proteins (especially Sec22) are involved in membrane fusion, they localize to ER and GA and help in anterograde and retrograde transport of vesicles. Anterograde transport is from ER to GA, cargo vesicles are formed in ER and coated with COPII. COPII coated vesicles interact with GA resulting in membrane fusion and vesicular components exchange. Retrograde transport is from GA to ER, cargo vesicles with ER resident move in the opposite direction from GA to ER and are coated in COPI. During retrograde transport, cargo proteins are budding off of the vesicles from GA or ERGIC. Sec22 is confined to ERGIC and can be replaced by Ykt6 during retrograde transport. Created with BioRender.com

SNARE proteins are not only involved in synaptic vesicle release and neuronal communication, but also in transport of hormones, growth factors, and other molecules within cells (Dhara et al., 2018; Gaisano, 2017; Südhof & Rothman, 2009). Cellular trafficking underlies many critical functions (Figure 3) and is vital to ensuring that transport vehicles reach their correct destination and that their contents are released in a temporally and spatially controlled manner. Cellular trafficking components are also involved in basic cellular functions, such as cell migration, cell division, secretion of insulin and other hormones in the body, and nutrient uptake. Therefore, defects in SNARE constituents, which are members of the family of cellular trafficking molecules, can lead to various conditions, both within and outside of the central nervous system.

### 1.2 Snap25

Synaptosomal‐associated protein of 25 kDa (Snap25) is found in three isoforms in neurons, namely Snap23, which is also expressed in nonneuronal cells, Snap25a and Snap25b. In cultured neurons, deletion of Snap25 leads to a reduction in survival, arborization, and spontaneous vesicle release, and complete elimination of evoked synaptic vesicle release (Bronk et al., 2007; Delgado‐Martínez et al., 2007; Washbourne et al., 2002). This phenotype can be rescued by any of the three isoforms, except for evoked synchronous synaptic vesicle release which can only be compensated for by Snap25a or Snap25b. Of the latter two, Snap25b has a higher potency at promoting vesicle priming (Delgado‐Martínez et al., 2007; Sørensen et al., 2003).

Loss of Snap25 in *vivo* results in abnormalities with species‐specific severity, probably because of different compensatory mechanisms by homologous proteins. For instance, in flies, deletion of Snap25 does not severely impair neurotransmission because of compensation by Snap24 (Niemeyer & Schwarz, 2000; Vilinsky et al., 2002). In mice, however, loss of Snap25 leads to lethality at birth and strong reduction of evoked synaptic vesicle release (Tafoya et al., 2008; Washbourne et al., 2002), in agreement with the absence of evoked vesicle release observed in *vitro*.

The effect of alterations of Snap25 function on postnatal development was studied in another mouse model, the Blind drunk (Bdr) mouse, which has a spontaneous missense mutation in Snap25b, unlike the Snap25 knockout mouse that has a complete abolition of the expression of all Snap25 protein isoforms. Bdr animals show impaired synaptic vesicle transport but survive until adulthood and display behavioural abnormalities including ataxia and sensorimotor gating deficits (Jeans et al., 2007). This suggests that, at least in mice, Snap25b may not be critical during earlier stages of development, or its function might be compensated for by other isoforms when it is not present.

In *vitro* and conditional knockout experiments gave insight into the role of Snap25 in later stages of development. When Snap25 null thalamic explants were co‐cultured with wildtype cortical explants (from postnatal day (P)0‐3 mouse brains), thalamocortical axons grew into the cortex, extended to the region of layer 4 and started to develop branches normally (Molnár & Blakemore, 1991, 1999), suggesting that regulated synaptic vesicle release by the developing thalamus is not required for the formation of initial thalamocortical connections (Blakey et al., 2012).

Subsequent experiments in conditional knockouts where Snap25 expression was abolished in a selected population of cortical projection neurons by Cre‐recombinase mediated excision of exon 5a/5b, allowed access to developmental stages after birth in *vivo* (Hoerder‐Suabedissen et al., 2019). Abolishing the expression of Snap25 in layer 5 (Rbp4‐Cre), layer 6 (Ntsr1‐Cre), and layer 6b (Drd1a‐Cre) from around the time of birth demonstrated that Snap25 is not required for the later stages of axonal targeting, branching, initiation of myelination or early synapse formation (Hoerder‐Suabedissen et al., 2019; Korrell et al., 2019).

**FIGURE 4:**
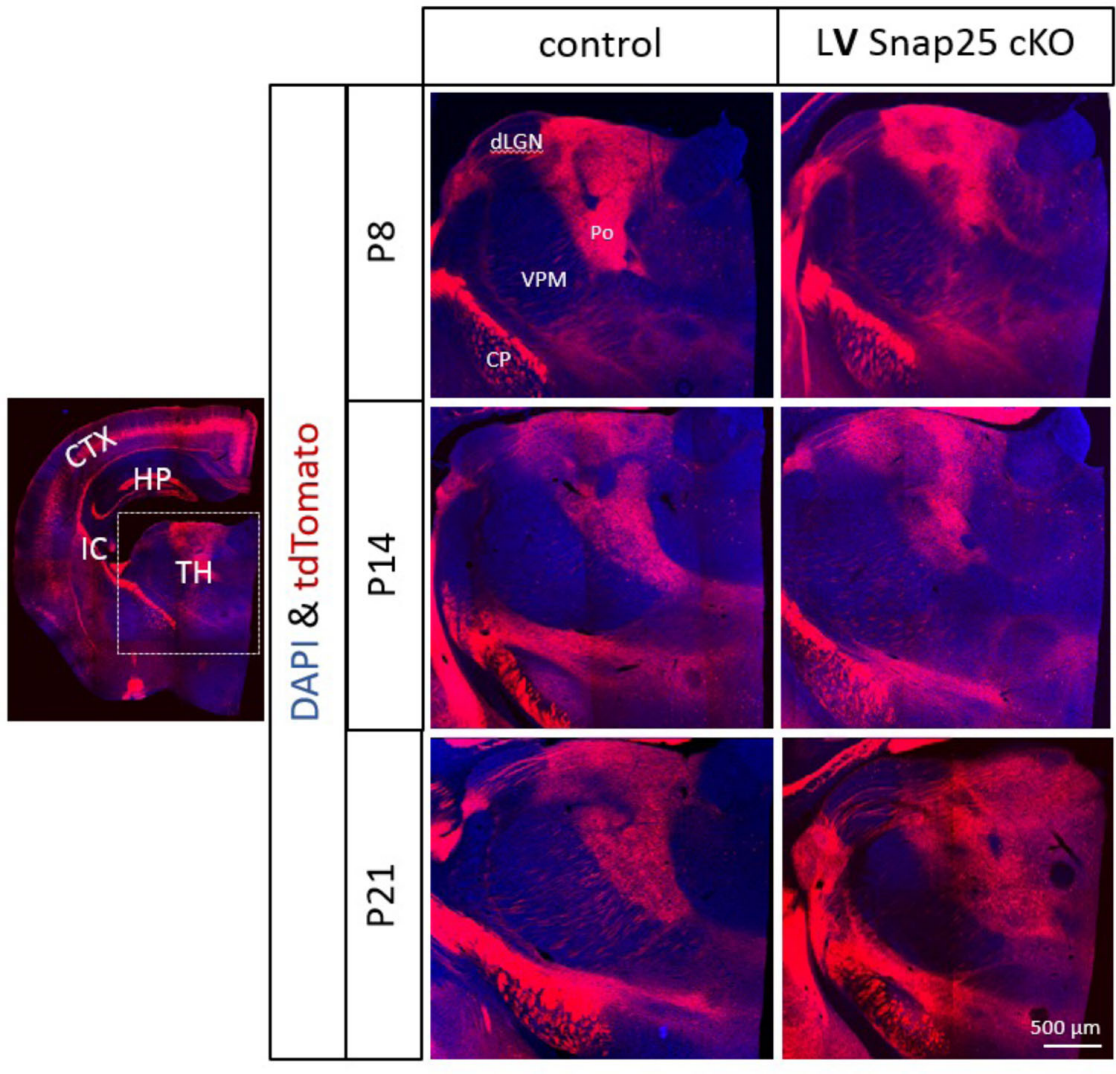
Normal projection of silenced layer 5 neurons Rbp4‐Cre;Ai14;*Snap*25^fl/fl^. in *vivo* Cre‐expression allows normal development of long‐range axonal projections in control and “silenced” Rbp4‐Cre expressing cortical L5 projection neurons. Images of *Rbp4‐Cre;Ai14;Snap25^fll+^* (control) or *Rbp4‐Cre;Ai14;Snap25^fl/fl^* brains (LV Snap25 cKO). (Upper row) Images of P8 brains from *Rbp4‐Cre;Ai14;Snap25^fll+^* or *Rbp4‐Cre;Ai14;Snap25^fl/fl^* mice, demonstrating presence of tdTom+ fibers projecting subcortically through the internal capsule (IC) and into the thalamus (TH). The boxed region of the left image is enlarged at P8, P14 and P21. Images of tdTom+fibers in the thalamus and cerebral peduncle at P8. By this time point, tdTom+ axons course through dorsal lateral geniculate nucleus (dLGN) and form dense terminal arborizations in Po and lateral posterior nucleus (LP). The subcerebral projections extend within the cerebral peduncle. The pattern, intensity and fasciculation patterns appear identical in the *Rbp4‐Cre;Ai14;Snap25^fll+^* and *Rbp4‐Cre;Ai14;Snap25^fl/fl^* brains. Structures normally devoid of L5 fibers such as habenula do not receive inappropriate innervation in the *Rbp4‐Cre;Ai14;Snap25^fl/fl^* brain. The pattern observed at P14 and P21 are similar and indistinguishable between *Rbp4‐Cre;Ai14;Snap25^fll+^* or *Rbp4‐Cre;Ai14;Snap25^fl/fl^*. Scale bars = 500 µm. CP, cerebral peduncle; dLGN, dorsal lateral geniculate nucleus; Hb, habenula; LP, lateral posterior nucleus; Po, posterior nucleus; VPM, ventral posterior medial nucleus. Data from Hoerder‐Suabedissen et al. (2019). https://academic.oup.com/cercor/article/29/5/2148/5025424?login=true

**FIGURE 5:**
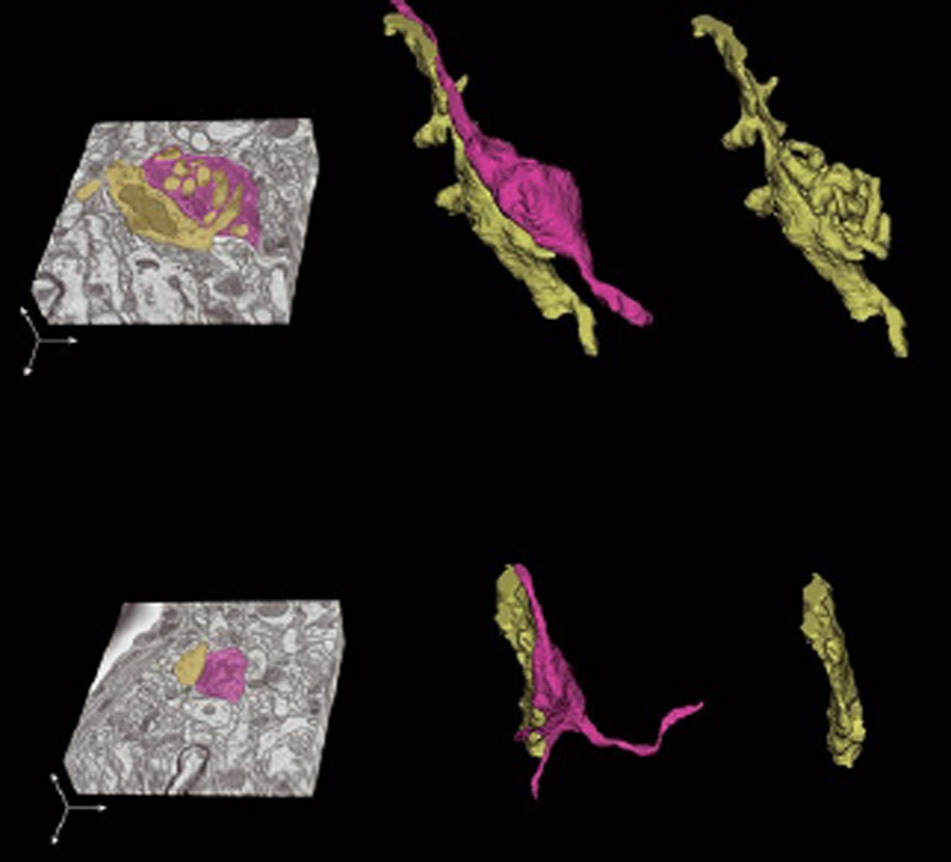
Lack of specialised synapse formation in PO, Rbp4‐Cre;Snap25^fl/fl^ boutons lack excrescences from Po dendrites at P18. Cross‐sectional view (left) and 3D reconstruction (middle and right) of an axon from cortical layer 5 (magenta) and its connecting dendrite (yellow) in the stack volume taken with serial block face scanning electron microscopy of the mouse posterior thalamic nucleus in a wild‐type (upper panel) and a Snap25 conditional knockout (lower) brains at P18. There are excrescences on the contact surface of the dendrite in the wild type brain (upper right), but not in the Snap25 cKO brain (lower right). See Hayashi et al. (2021). Maturation of complex synaptic connections of layer 5 cortical axons in the posterior thalamic nucleus requires Snap25. From Hayashi et al. (2021) (https://academic.oup.com/cercor/article/31/5/2625/6047731?login=true)

Cortical layer 5 projections target higher order thalamic nuclei in the thalamus and form specialized synapses with thalamic neurons (Grant et al., 2016; Hoerder‐Suabedissen et al., 2019) (Figure 4). Normally, the target thalamic neurons extend their dendritic protrusions into the layer 5 axon's giant boutons (Groh et al., 2008; Hoogland et al., 1991). However, when Snap25 expression is abolished specifically in layer 5 neurons from birth, these specialized synapses between the giant terminals of the cortical axons with the posterior thalamic neurons show altered development, with the thalamic dendritic protrusions failing to mature into the giant interdigitating shape. This suggests that Snap25‐mediated synaptic vesicle release by cortical layer 5 neurons is not crucial for initial formation of synapses but is fundamental for the maturation of specialized synapses (Hayashi et al., 2021) (Figure 5).

### 1.3 Munc13 and Munc18

The Munc13 (*encoded by the UNC13A gene in humans*) protein family consists of four genes (*Munc13‐1, 2, 3, 4*) (Brose et al., 1995; Calloway et al., 2015; Koch et al., 2000; Südhof, 2012) which are all highly expressed in the cortex, hippocampus, cerebellum, striatum, basolateral amygdala, and olfactory bulb (Augustin et al., 2001, 1999; Gioia et al., 2016). To form the SNARE complex, Munc13 facilitates opening of syntaxin‐1 (Ma et al., 2011) and is necessary for spontaneous synaptic vesicle release, a process known as synaptic vesicle priming (Varoqueaux et al., 2002), fusion (Augustin et al., 1999), neurotransmitter release (Chen et al., 2013; Quade et al., 2019; Varoqueaux et al., 2002); and plays a role to form short‐term plasticity (Chen et al., 2013; Zikich et al., 2008).

Another important protein required for synaptic vesicle fusion is Munc18‐1, also known as syntaxin‐binding protein‐1. Homologs of Munc18 (*encoded by the STXBP1 gene in humans*), known as sec1/Munc18‐like proteins or SM proteins, are important for intracellular fusion. Munc18‐1 is essential for all transmitter release, but early developmental synaptogenesis is not altered in its absence (Verhage et al., 2000). Most SM proteins interact with members of the syntaxin protein family. Munc18‐1 interacts with SNARE by two different ways and it either binds to closed syntaxin‐1 in a binary complex or it binds to SNARE complex proteins in heteromultimeric assembly (Dulubova et al., 1999) (Figure 2). Furthermore, Munc13 and Munc18 together prevent de‐priming of synaptic vesicles (He et al., 2017) and act as chaperons by coordinating a proper SNARE complex assembly. However, it remains unclear how Munc13 and 18 are involved in normal neuronal network formation.

### 1.4 Munc13

Proteins of the Munc13 family have been studied in different in *vivo* and In *vitro* models that demonstrated their role in normal neuronal network formation and maintenance, and their role in various neurodevelopmental and neurodegenerative diseases. Neurons lacking both Munc13‐1 and Munc13‐2 isoforms have neither evoked nor spontaneous activity, yet they form synapses with a typical structure In *vitro* (Varoqueaux et al., 2002).

Mice lacking Munc‐13 have impaired synaptogenesis and are born paralyzed (Varoqueaux et al., 2005). In the ribbon synapses of the adult mouse retina, lack of Munc13‐2 has little effect on synaptic signaling (Cooper et al., 2012; Zikich et al., 2008). In contrast, the Munc13‐2 isoform is necessary for normal release probability required for plasticity in mouse hippocampal mossy fiber synapses (Breustedt et al., 2010). The formation and maintenance of functional synapses and synaptic spines have been demonstrated in the absence of presynaptic glutamate release (Sigler et al., 2017; Sando et al., 2017). In the cerebellum, mice lacking the Munc13‐3 isoform have higher paired‐pulse facilitation and impaired motor task learning (Augustin et al., 2001). Moreover, Munc13‐3 null mutant mice show decreased synaptic probability in cerebellar neurons and have a strong decrease in the acoustic startle response (Netrakanti et al., 2015). These studies suggest that Munc13‐mediated vesicle priming is isoform and synapse specific.

### 1.5 Munc18

The Munc18‐1 isoform has multiple roles in synaptic vesicle exocytosis, and it promotes the stability of syntaxin. Guiberson and colleagues showed that expressing mutant forms of Munc18‐1 identified in human disease in the knock out of endogenous (WT) Munc18‐1 affect neurotransmitter release and vesicle recycling (Guiberson et al., 2018).

Munc18‐1 is spliced into long (M18L) and short (M18S) isoforms that are critical for the modulation of neurotransmission (Ramos‐Miguel et al., 2015). The Munc18‐1 long splice variant is localized to the synaptosomal fraction in GABAergic terminals, and the short variant in cytosol and synaptosomal compartments in both GABAergic and glutamatergic terminals (Ramos‐Miguel et al., 2015). Transgenic mice with erased M18L show severe physical and behavioural dysfunction and die within a few weeks after birth.

Munc18‐1 deficient mice have defective transmission in the neuromuscular junction of heart and lungs, which leads to perinatal lethality. However, heterozygous Munc18‐1 null mutant mice survive, although the release of acetylcholine at neuromuscular junction is reduced (Sons et al., 2003). The lack of Munc18‐1 in serotonergic neurons causes immediate degeneration of the 5‐HT system as well as postnatal lethality in mice (Dudok et al., 2011). These studies revealed that mutations of Munc18‐1 affect normal stability of different types of synapses thus leading to impaired neuronal network maintenance.

Bouwman and colleagues demonstrated normal initial synapse formation but then dramatic loss of synapse number in cortex in Munc18‐1 KO mice (Bouwman et al., 2004). They reported that reduction in synapse is not due to apoptosis or degeneration and secretion of neurotransmitters and neuromodulatory substances is required for synapse maintenance, not for early synaptogenesis. A subsequent study suggested that Munc18‐1 has an early role in the Golgi organelle (Santos et al., 2017). Heterozygous mutatioons in Munc18‐1 cause early infantile epileptic encephalopathies in humans, probably as a result of excitation‐inhibition unbalance (Kovacevic et al.,).

### 1.6 Syntaxin

Syntaxins are transmembrane proteins involved in regulating intracellular membrane trafficking, membrane fusion events, and the specificity of transport vesicle docking and fusion (Duman & Forte, 2003; Yoon & Munson, 2018). Of the 15 mammalian syntaxins described so far, syntaxin 1A, 1B, 2, 3, and 4 reside on the plasma membrane and the spliced isoforms of syntaxin1 have been shown to be confined to the presynaptic membrane and to be implicated in neural exocytosis (Lang & Jahn, 2008; Teng et al., 2001). The involvement of syntaxins in mediating postsynaptic exocytosis and neurotransmitter receptor trafficking has also been supported with shRNA approaches to knock down different components of the SNARE machinery.

In thalamocortical cultures, specific cleavage of syntaxin 1A by botulinum toxin C1 impacts the uptake of serotonin and diminishes the number of cell surface serotonin transporters. By changing the intracellular distribution of these transporters, syntaxin 1A might affect early cortical development (Quick, 2002). Moreover, syntaxin 1A interacts with other neurotransmitter transporters including the glycine transporters GLYT1 and GLYT2 and the GABA transporter GAT1, which are important in cortical development (Deken et al., 2000).

Loss of function studies on the isoforms of syntaxins have been hindered by the lack of appropriate knockout animal models and embryonic lethality. Syntaxin 1a/1b double knockout embryonic brains show compensatory changes in the form of decreased expression levels of various other synaptic proteins including Synaptobrevin‐2, Munc13‐1, Snap25, complexin‐1, and Rab5 (Mishima et al., 2014; Vardar et al., 2016). In this mouse model, early cytotoxicity is seen, which indicates the importance of Syntaxin1 in neuronal survival in early development (Verhage et al., 2000).

### 1.7 Synaptobrevins/VAMPs

Synaptobrevins or vesicle‐associated membrane proteins (VAMPs) are other components of the synaptic machinery and have been knocked out in various mouse models. Homozygous deletion of synaptobrevin‐2/VAMP2 results in perinatal lethality in mice, and the mutants have a noticeably different body shape including a shoulder hump (Schoch et al., 2001) that might be attributed to defects in exocytic events in endocrine cells and adipocytes. On the other hand, a mere reduction in VAMP2 protein levels does not cause morphological abnormalities and brain structures are intact (Raptis et al., 2005; Schoch et al., 2001). Some studies propose a role for synaptobrevin‐2/VAMP2 in axonal guidance (Zylbersztejn et al., 2012). E18 embryos from Synaptobrevin‐2/VAMP2 null mice showed defasciculating Nrp1‐expressing axonal fibers in the corpus callosum (Piper et al., 2009; Zylbersztejn et al., 2012). VAMPs may also play a role in regulation of sleep‐wake states, as VAMP2 mutants exhibit a sleep phenotype characterized by a substantially reduced propensity in switching vigilance states with an especially pronounced deficit in initiating rapid eye movement (REM) sleep episodes (Banks et al., 2020).

### 1.8 SNARE complex in interactions between glial cells

#### 1.8.1 Roles of the SNARE complex in myelination

The SNARE complex is necessary not only for neuron–neuron communication but also neuron–glia and glia–glia communication, and plays critical roles in conduction velocity by modulating myelination as well as synaptic maturation and plasticity.

Myelin is a lipid–protein complex synthesized by mature oligodendrocytes in the central nervous system (CNS) of vertebrates. Myelin insulates most nerve fibers, and the myelin sheath contains periodic breaks known as nodes of Ranvier that facilitate fast, saltatory conduction of neural signals. Interestingly, brain activity may modulate myelination (Banks et al., 2020; Hines et al., 2015). in *vivo* studies revealed that myelinating cells have a high expression of neurotransmitter receptors (Butt et al., 2014; Micu et al., 2006), and calcium activity (Micu et al., 2007) suggesting activity‐dependent myelination. The importance of SNARE complex protein expression within oligodendrocytes and within neurons have both been demonstrated for myelination.

#### 1.8.2 Oligodendrocytes and Schwann cells in myelination

**FIGURE 6:**
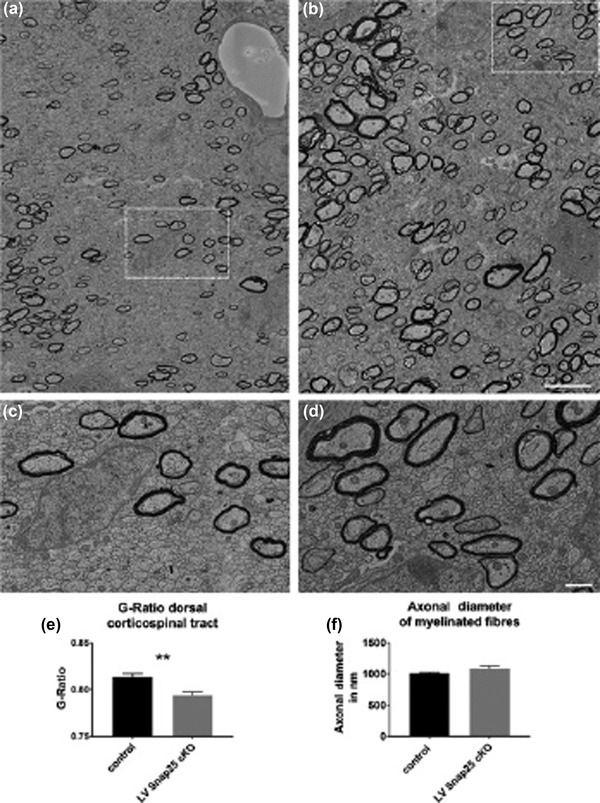
Maintenance of the myelin is reduced with reduced g‐ratio in LV Snap25 cKO dorsal spinal columns despite normal axon diameter. (a) Tiled scanning electron microscope (SEM) image of the g‐ratio quantification region of one side of the dorsal spinal column in LV control mice at P18. A higher magnification view of the boxed region is shown in (c). (b) Tiled SEM image of the g‐ratio quantification region of one side of the dorsal spinal column in LV Snap25 cKO spinal cord at P18. A higher magnification view of the boxed region is shown in (d). (e) The average g‐ratio of LV Snap25 cKOs (*n* = 4) shows a significant reduction in comparison to the g‐ratio of controls (*n* = 5, *p* = .0072). (F) Axonal diameter of myelinated fibres was not significantly different between controls and LV Snap25 cKOs. Scale bars: 5 µm (a,b) 1 µm (c,d). Figure from Korrell et al. (2019) https://onlinelibrary.wiley.com/doi/full/10.1111/joa.12974

Myelination by oligodendrocytes requires trafficking of myelin membrane components. It has been shown that the SNARE complex proteins VAMP3 and VAMP7 are involved in this trafficking process, as they colocalize with myelin proteolipid protein (PLP) in recycling and late endosomes (Feldmann et al., 2011). RNA‐mediated silencing of VAMP3 or VAMP7 diminishes trafficking of PLP to oligodendrocytes (Feldmann et al., 2011). Moreover, AP‐3δ‐deficient *mocha* mice which have impaired secretion of lysosome‐related organelles and missorting of VAMP7, show mild dysmyelination that can be explained by the reduced level of PLP (Feldmann et al., 2011). VAMP2‐deficient mice, on the other hand, do not show any changes in myelination. Both VAMP3 and VAMP7 are necessary for vesicular trafficking, especially VAMP7, which contributes to myelination by transporting cargo vesicles to the myelin membrane in the CNS (Feldmann et al., 2011). However, abolishing expression of Snap25 in selected subpopulations of cortical layer 5 and 6 projection neurons have effect on maintenance of myelination but not the onset of myelination (Korrell et al., 2019; Figure 6).

In the peripheral nervous system, myelination is performed not by oligodendrocytes but by Schwann cells. Disruption of synaptic vesicle release in the Munc13‐1/2 double knockout mouse model led to an increase in the number of Schwann cell bodies in the spinal cord, although no effect on axon myelination was observed (Varoqueaux et al., 2005). In zebrafish, tetanus toxin (TeNT) was used to block VAMP2‐mediated synaptic vesicle release (Verderio et al.,). At early larval stages, this led to a decrease in the number of myelinated axons in the spinal cord as well as a reduction in the number of myelin sheaths per oligodendrocyte (Verderio et al., 1999).

#### 1.8.3 Neuron–glia interactions in myelination

Besides its role in regulating synaptic transmission, the SNARE complex is also essential for the secretion of hormones and growth factors required for myelination such as brain‐derived neurotrophic factor (BDNF). The levels of BDNF increase during the first 3 weeks after birth (Andreska et al., 2020) which suggests its developmental role in axonal growth, synaptogenesis, and synaptic maturation. BDNF increases the expression of myelin proteins in oligodendrocytes via activation of the tropomyosin‐related receptor kinase‐B/mitogen‑activated protein kinase (TrkB/MAPK) pathway (Xiao et al., 2009) that is critical for the increase in thickness of myelin, but not for the initiation of myelination (Ishii et al., 2019; A. W. Wong et al., 2013). Deletion of Snap25 in layer 6 predominantly reduces maintenance of myelination (Korrell et al., 2019). As BDNF is expressed in pyramidal neurons in layers 2/3, 5 and 6 and presents in some of the cortex‐derived afferents and presynaptic terminals in the dorsolateral striatum (Andreska et al., 2020; Conner et al., 1997), BDNF might be responsible for the developmental defects in the maintenance of myelin in the layer‐specific Snap25 conditional knockout mice. Therefore, less activation of the TrkB/MAPK signaling might be an explanation for the impaired myelination seen in this mouse model (Figures 6).

#### 1.8.4 Roles of the SNARE complex in astrocytes

Astrocytes are known to secrete many factors to promote synapse formation and functioning (Schubert et al., 2011; Wilhelm et al., 2004). They contain several secretory vesicles including synaptic‐like microvesicles (SLMV) and dense‐core vesicles (DCV), which enclose glutamate and d‐serine, and ATP and BDNF, respectively. Similar to other nonneuronal cells, the SNARE complex in astrocytes consists mainly of Snap23, syntaxin 4, and synaptobrevin‐2/VAMP2 and cellubrevin/VAMP3 (reviewed in Mielnicka & Michaluk, 2021). A recent study shows that astrocytic VAMP3‐dependent secretion of neuropeptide Y reduces synaptic signaling while VAMP2–dependent glutamate release from astrocytes enhances the signaling (Schwarz et al., 2017). VAMP3 also selectively regulates the release of endocytic (recycled) BDNF from astrocytes (Han et al., 2021). Moreover, astrocytic VAMP2‐mediated release of thrombin protease inhibitors at the node of Ranvier modulates myelin sheath thickness and nodal gap length in the optic nerve (Dutta et al., 2018). The function of SNARE proteins in astrocytes is still not well known and there is a need for better understanding in the future.

#### 1.8.5 Roles of the SNARE complex in microglia

Microglial cells help to maintain a stable and healthy brain environment and play a key role in shaping neuronal network formation during development (Wake et al., 2013). After birth, brains contain overabundant neuronal connections that must be refined and eliminated to secure the most effective and efficient brain activity (LaMantia & Rakic, 1990; Miyamoto et al., 2016; Seeman et al., 1987). These cells act as a gardener and scavenge unnecessary connections in a process known as synaptic pruning. This process helps to shape a neuronal network that is systematically organized to subserve its complex functions (Seeman et al., 1987). One of the models of synaptic pruning suggests that neuron–microglia interactions may be activity‐dependent. Microglia can generate glutamate neurotransmitters as well as respond to any neurotransmission changes by changing morphology (McMullan et al., 2012; Noda et al., 2000). Microglia stimulation with ionomycin indicates that microglia release ATP and in a calcium and SNARE‐dependent manner (Imura et al., 2013). It was also indicated that microglial cells express the Snap25 analogue Snap23 involved in phagocytic processes (Hepp et al., 1999; Rojewska et al., 2018). Moreover, overexpression of Munc18 reduces the number of cortical microglia in the mouse brain (Rojewska et al., 2018).

**FIGURE 7:**
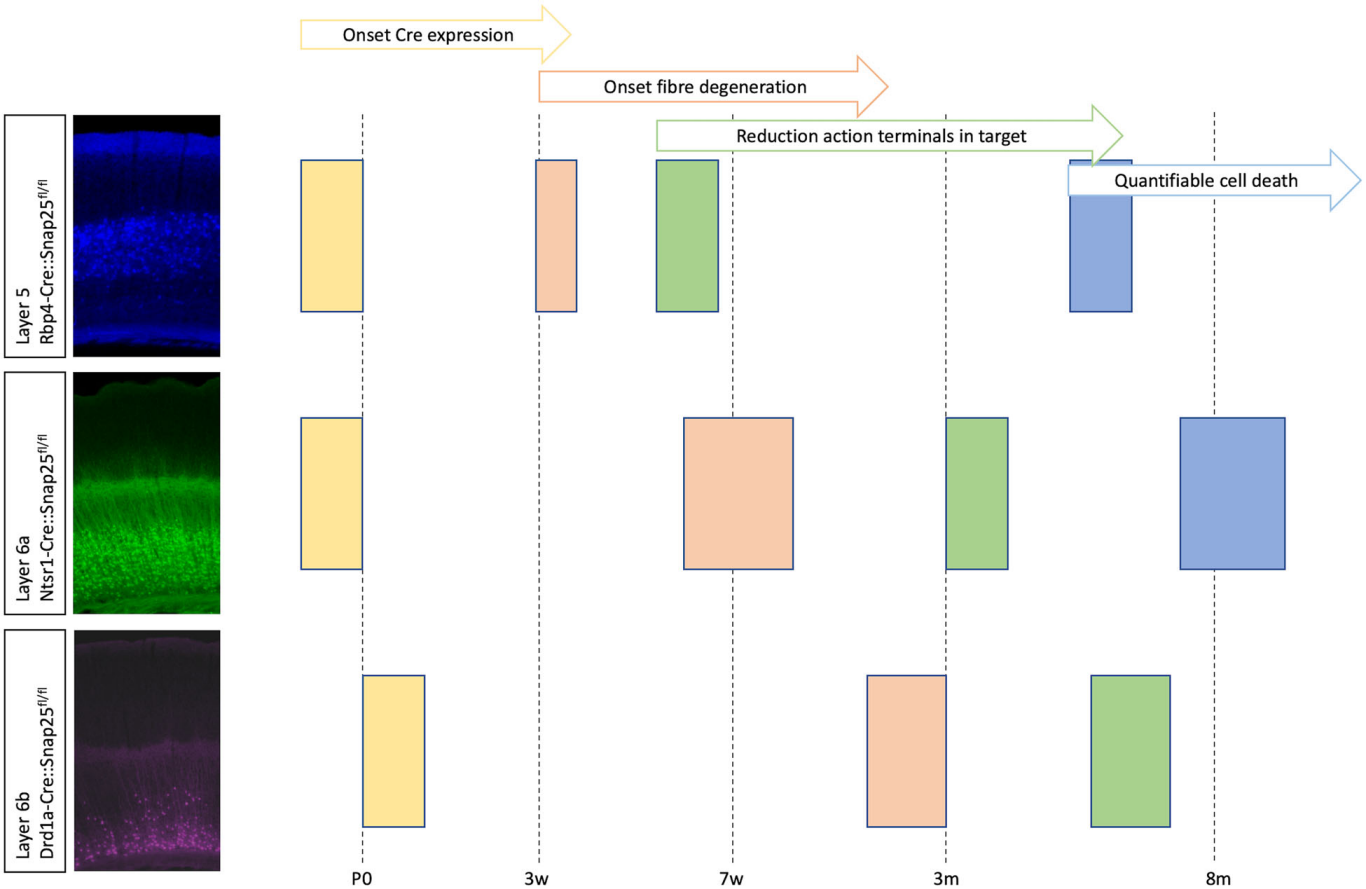
Degenerative processes following genetic ablation of Snap25 in different cortical projection neurons using mouse Cre lines. Representative fluorescent images of reporter gene expression in cortex of Rbp4‐Cre;Snap25fl/fl (p36), Ntsr1‐Cre;Snap25fl/fl (p48) and Drd1a‐Cre;Snap25fl/fl (p21) mice. Cre‐recombinase expression starts during late embryonic or early postnatal life in the respective strains. Fragmentation of distal axons is detectable from P22 in Rbp4‐Cre cKO brains, but not until 2 months of age in Ntsr1‐Cre conditional knockout (cKO) and 3 months in Drd1a‐Cre cKO brains. A decrease in the density of terminal arbours in subcortical target structures is evident by P28 for Rbp4‐Cre cKO brains but decreases only become detectable after 3 months of age for Ntsr1‐Cre cKO and 5 months of age in Drd1a‐Cre cKO brains. A reduction in the density of cortical Cre+ neurons was evident for both Rbp4‐Cre and Ntsr1‐Cre cKO brains around 8–10 months of age, but no such reduction could be demonstrated in Drd1a‐Cre cKO brains over the time period studied. Data from Hoerder‐Suabedissen et al. (2019). https://academic.oup.com/cercor/article/29/5/2148/5025424?login=true

Microglial crosstalk with oligodendrocytes plays a crucial role in myelination. During postnatal development, overproduced myelin sheaths are selectively eliminated by microglia (Hughes & Appel, 2020; Irfan et al., 2021). Following neuronal silencing with either dominant‐negative VAMP2 (dnVAMP2) or botulinum toxin (BoNT/B), microglial phagocytosis of the myelin in the optic tectum and spinal cord of zebrafish is enhanced (Hughes & Appel, 2020). Enhancement of microglial phagocytic capacity might result in impaired myelination during postnatal development in layer 6‐specific Snap25 conditional knockout mice and axonal degeneration in layer 5‐specific Snap25 conditional knockout adult mice (Figure 7). Interestingly, these silenced neurons fail to be maintained, with a degeneration that appears at about 4 weeks for the layer 5 and at around 6 weeks for the layer 6 projection neuron (Korrell et al., 2019). The expression levels of phagocytic ligands and receptors, for example, C1q, fractalkine, UDP, Fc‐receptor gamma and scavenger receptor‐AI/II, might be changed in the layer‐specific neural silencing models. Furthermore, as neuronal BDNF prevents microglial phagocytosis of synapses in hippocampal neurons (Onodera et al., 2021), the altered levels of BDNF might be a possible key factor for the impaired myelination and axonal degeneration. However, it remains unclear how microglia are involved in synaptic vesicle release and trafficking under normal conditions, and whether there is direct or indirect interaction with SNARE complex proteins.

### 1.9 SNARE pathology in neuropsychiatric and neurodegenerative disorders

Regulated and spontaneous synaptic vesicle release plays an essential role in the formation and maturation of synapses. Changes in SNARE function can lead to synaptic pathology and altered neuronal function and can result in neurodevelopmental, neuropsychiatric, and neurodegenerative diseases. Although many of these diseases are multi‐genetic, abnormalities in specific SNARE complex components function can be distinguished in some of them and have also been used to model these disorders.

### 1.10 Neurodevelopmental and neuropsychiatric disorders

Human studies revealed that the homozygous N‐terminal stop‐codon mutation in Munc13‐1 has abnormal cortical activity and is linked with microcephaly (Engel et al., 2016). Mutations of Munc18‐1 are linked to various disorders such as epileptic encephalopathies (Tang et al., 2021), motor function disorders (Miyamoto et al., 2015) and neurodevelopmental disorders (Tang et al., 2021). Moreover, due to the same mutation metabolic stability is impaired and rapid protein degradation of Munc18‐1 can be one of the underlying causes of Munc‐18‐1‐related encephalopathies.

The chromosomal location of the HCP‐1/ Syntaxin1A gene (7q11.23) corresponds to the region typically deleted in Williams Beuren syndrome and the deletion of the syntaxin1‐A gene has been proposed to underlie certain neurological and cognitive deficits of this neurodevelopmental disorder such as impaired verbal, visuospatial, memory, and numerical skills (Fusco et al., 2013; Herwegen, 2015).

Overexpression of the *Munc18‐1* gene has been described in patients with schizophrenia and studies with mice overexpressing M18L show a schizophrenia‐like phenotype (Urigüen et al., 2013). These findings indicate that the presence of the M18L variant in inhibitory terminals is associated with cognitive function and implies that M18L level changes could be related with cognitive disorders.

Similarly, the sensorimotor gating deficits observed in Bdr mice can be explained as a schizophrenia‐like phenotype, as similar abnormalities in pre‐attentive processing have been observed in human patients with schizophrenia, linking Snap25 activity to cognitive functioning (Jeans et al., 2007). Moreover, in a major mouse model of schizophrenia, in which a human variant of the disrupted in schizophrenia (DISC1) gene is expressed transgenically, the hDISC1 mouse, Snap25 levels are concomitantly decreased. This model shows similarities to the negative symptoms of schizophrenia, including altered social interaction, anxiety, and reduced spatial memory supporting the translational applicability of this mouse model (Pletnikov et al., 2008). These results imply that Snap25 expression and thus regulated synaptic vesicle release is important for neurodevelopment and its disruption can lead to neuropsychiatric symptoms later in life.

Changes in the expression of Snap25 have also been associated with attention‐deficit hyperactivity disorder (ADHD). In the *Coloboma* mouse, there is a 50% reduction in Snap25 expression, and the mice show a phenotype of increased locomotor activity during the nocturnal, active phase, which has similarities to the behavior observed in children with ADHD (Hess et al., 1996, 1992). Association of SNAP25 polymorphisms with ADHD in human patients has also been confirmed in a meta‐analysis (Faraone et al., 2005). The problem with the Coloboma mouse is that many other genes are affected alongside Snap25, making its phenotype difficult to interpret in isolation.

### 1.11 Neurodegenerative disorders

Munc13 isoform 1 can be an alternative target of the intracellular diacylglycerol second messenger pathway in Alzheimer's diseases and plays a role in the regulation of amyloid precursor protein metabolism (Rossner et al., 2004).

The loss of Snap25 from subsets of cortical layer 5, 6a, or 6b projection neuron populations, respectively has been shown to result in degeneration of some of these neurons in mice, including formation of autophagosome‐lysosome‐like structures, and increases in inflammatory markers. The time course of degeneration differs per driver line, suggesting a subpopulation‐specific specific necessity of Snap25 for neuronal survival (Hoerder‐Suabedissen et al., 2019) (Figure 7). Supporting a role of Snap25 in neuronal maintenance, knockout of the chaperone protein cystein rich protein alpha (CSP‐alpha) causes neurodegeneration in mice by impairing the function of Snap25, possibly mediated through proteasomal degradation (Sharma et al., 2012). Cell death caused by loss of Snap25 is correlated with a condensation of the cis‐Golgi system, different from Golgi fragmentation that is seen in apoptosis, suggesting that a different mechanism of neurodegeneration occurs when Snap25 activity is interrupted (Santos et al., 2017).

## 2 SUMMARY

In this review, we describe the role of various forms of synaptic activity in brain development, with special attention to the cerebral cortex. During the course of development, neuronal connections form, synapses get established and neurotransmitter release shifts from spontaneous to regulated. Paracrine secretion of neurotransmitters from growing neurites can influence axonal guidance and formation of new connections. It has recently become possible to study the specific role of spontaneous constitutive release of neurotransmitters and compare this with the spontaneous vesicular and regulated vesicular transmitter release in intercellular communication. This became possible with the discovery of molecular mechanisms and the generation of selective knockout of individual proteins in mouse models.

Selective knockout experiments have indicated that regulated synaptic vesicle release is not necessary for early development such as neurogenesis, neuronal migration, and early formation of connections and not even for initial formation of synapses. However, disruption of regulated vesicle release results in embryonic lethality because of its necessity in the neuromuscular junction of respiratory and other muscles.

The conditional ablation of Snap25 has allowed us to interfere with regulated synaptic vesicle release in selected neural populations, preserving the function of the rest of the brain and preventing embryonic lethality. In these conditional knockout models, the initial axon outgrowth, targeting, myelination, synapse formation was not affected, however the maintenance of synapses, myelin, and formation of specialized synapses was disturbed. Lack of Snap25 eventually led to loss of synapses, myelin, axons, and death of some of the projection neurons, with a time course that was determined by the neural population targeted in our conditional knockout. Generation of conditional knockout for other SNARE complex members, such as Munc13, will enable us to explore the effects of absence of both regulated and spontaneous synaptic vesicle release on neurodevelopment. These and other models will help to dissect the role of different forms of synaptic communication in specific phases and processes of development.

## 3 FUTURE DIRECTIONS

With the expansion of knowledge on the molecular mechanisms of synaptic vesicle release and the development of mouse transgenic technology, the respective contributions of different SNARE molecules in cortical development can now be studied in relative isolation. Moreover, by limiting knockout of genes to selected neural populations in Cre‐dependent models, the embryonic lethality that was commonly seen in SNARE knockouts can be circumvented, allowing investigation of processes in postnatal development such as myelination and neuronal maintenance.

A main confounder when studying the role of neural activity in a developmental process by means of intervening with SNARE activity, is the multipurpose nature of the SNARE complex. The SNARE complex is not solely involved in the release of neurotransmitter vesicles at the synapse, but also key for release of vesicles containing hormones and growth factors, such as insulin and BDNF (Shimojo et al., 2015). Therefore, it is difficult to determine if the abnormal maturation that is seen in conditional knockout models is the result of the absence of neurotransmitter release or the absence of growth factor release or both.

Snap25 seems to influence various processes in development, but underlying mechanisms require further investigation. For instance, Snap25 seems to influence myelination as its absence leads to myelination abnormalities (Korrell et al., 2019) but how Snap25 is involved in this process is not known. More precise temporal regulation of the Snap25 ablation could reveal how shorter or longer, and early or late ablation would influence the formation and maintenance of myelin. Similarly, further studies are needed to investigate how Snap25 conditional knockout causes axonal degeneration, and whether this degeneration is a result of the early developmental defects, or of a process independent of these abnormalities.

Most currently examined conditional knockout models have chronic effects, with ablation of the synaptic proteins from onset of Cre expression perinatally. Adding an additional regulatory element with a Tet‐On/Off system would give greater temporal resolution of genetic intervention, allowing the study of the role of specific proteins in selected processes of development. Additionally, the neuronal activity in a cell type of interest can now be manipulated in a reversible manner, using chemogenetics or optogenetics. For instance, the effects of chronic synaptic silencing of specific neuronal populations by ablation of Snap25 can be compared with the effects of acute neuronal silencing by expression of inhibitory designer receptors activated by designer drugs (DREADDs) or inhibitory optogenetic channels such as archaerhodopsin (ArchT). The difference between chronic and acute silencing can then be explained by the effects of regulated synaptic activity in the developing versus in the adult brain.

The selective ablation of Snap25 in different populations of cortical projection neurons resulted in different timelines of synaptic loss, demyelination, and axonal degeneration suggesting that the development and maintenance of these different cortical projection neuronal populations might be influenced differentially by mechanisms that require Snap25. It would be interesting to control spatial expression of Snap25 with even greater specificity and to investigate whether its presence is necessary for vesicular release from axonal shafts or from synapses.

Remarkably, the effects of conditional knockout of Snap25 are less severe in *vivo* than In *vitro*. This suggests that there might be compensatory mechanisms in play that can reduce the impact of a loss of Snap25. What these mechanisms involve and whether they are also activated when spontaneous abnormalities in Snap25 function occur is a question for future studies.

## CONFLICT OF INTEREST

The authors declare no conflict of interest.

## References

[dneu22892-bib-0001] Andreae, L. C. , & Burrone, J. (2018). The role of spontaneous neurotransmission in synapse and circuit development. Journal of Neuroscience Research, 96(3), 354–359. 10.1002/jnr.24154 29034487 PMC5813191

[dneu22892-bib-0002] Andreae, L. C. , & Burrone, J. (2015). Spontaneous neurotransmitter release shapes dendritic arbors via long‐range activation of NMDA receptors. Cell reports, 10(6), 873–882. 10.1016/j.celrep.2015.01.032 25683710 PMC4542315

[dneu22892-bib-0003] Andreae, L. C. , Fredj, N. B. , & Burrone, J. (2012). Independent vesicle pools underlie different modes of release during neuronal development. Journal of Neuroscience, 32(5), 1867–74. 10.1523/jneurosci.5181-11.2012 22302825 PMC6703344

[dneu22892-bib-0004] Andreska, T. , Rauskolb, S. , Schukraft, N. , Lüningschrör, P. , Sasi, M. , Signoret‐Genest, J. , Behringer, M. , Blum, R. , Sauer, M. , Tovote, P. , & Sendtner, M. (2020). Induction of BDNF expression in layer II/III and layer V neurons of the motor cortex is essential for motor learning. Journal of Neuroscience, 40(33), 6289–6308. 10.1523/JNEUROSCI.0288-20.2020 32651187 PMC7424868

[dneu22892-bib-0005] Augustin, I. , Korte, S. , Rickmann, M. , Kretzschmar, H. A. , Südhof, T. C. , Herms, J. W. , & Brose, N. (2001). The cerebellum‐specific Munc13 isoform Munc13‐3 regulates cerebellar synaptic transmission and motor learning in mice. Journal of Neuroscience, 21(1), 10–7. 10.1523/jneurosci.21-01-00010.2001 11150314 PMC6762458

[dneu22892-bib-0006] Augustin, I. , Rosenmund, C. , Südhof, T. C. , & Brose, N. (1999). Munc13‐1 is essential for fusion competence of glutamatergic synaptic vesicles. Nature, 400(6743), 457–61. 10.1038/22768 10440375

[dneu22892-bib-0007] Banerjee, A. , Barry, V. A. , DasGuptat, B. R. , & Martin, T. F. J. (1996). N‐ethylmaleimide‐sensitive factor acts at a prefusion ATP‐dependent step in Ca2+‐activated exocytosis. Journal of Biological Chemistry, 271(34), 20223–20226. 10.1074/jbc.271.34.20223 8702750

[dneu22892-bib-0008] Banks, S. M. L. , Medeiros, A. T. , McQuillan, M. , Busch, D. J. , Ibarraran‐Viniegra, A. S. , Sousa, R. , Lafer, E. M. , & Morgan, J. R. (2020). Hsc70 Ameliorates the vesicle recycling defects caused by excess α‐synuclein at synapses. ENeuro, 7(1), 10.1523/ENEURO.0448-19.2020 PMC7031854 31941659

[dneu22892-bib-0009] Barrecheguren, P. J. , Ros, O. , Cotrufo, T. , Kunz, B. , Soriano, E. , Ulloa, F. , Stoeckli, E. T. , & Araújo, S. J. (2017). SNARE proteins play a role in motor axon guidance in vertebrates and invertebrates. Developmental Neurobiology, 77(8), 963–974. 10.1002/dneu.22481 28033683

[dneu22892-bib-0010] Bouwman, J. , Maia, A. S. , Camoletto, P. G. , Posthuma, G. , G, R. , E, W. , Oorschot, V. M. J. , Klumperman, J. , & Verhage, M. (2004). Quantification of synapse formation and maintenance in vivo in the absence of synaptic release. Neuroscience, 126(1), 115–26. 10.1016/j.neuroscience.2004.03.027 15145078

[dneu22892-bib-0011] Blakey, D. , Wilson, M. C. , & Molnár, Z. (2012). Termination and initial branch formation of SNAP‐25‐deficient thalamocortical fibres in heterochronic organotypic co‐cultures. European Journal of Neuroscience, 35(10), 1586–1594. 10.1111/j.1460-9568.2012.08120.x 22607004 PMC3359864

[dneu22892-bib-0012] Breustedt, J. , Gundlfinger, A. , Varoqueaux, F. , Reim, K. , Brose, N. , & Schmitz, D. (2010). Munc13‐2 differentially affects hippocampal synaptic transmission and plasticity. Cerebral Cortex, 20(5), 1109–20. 10.1093/cercor/bhp170 19700493

[dneu22892-bib-0013] Bronk, P. , Deák, F. , Wilson, M. C. , Liu, X. , Südhof, T. C. , & Kavalali, E. T. (2007). Differential effects of SNAP‐25 deletion on Ca2+‐dependent and Ca2+‐independent neurotransmission. Journal of Neurophysiology, 98(2), 794–806. 10.1152/jn.00226.2007 17553942

[dneu22892-bib-0014] Brose, N. , Hofmann, K. , Hata, Y. , & Sudhof, T. C. (1995). Mammalian homologues of Caenorhabditis elegans unc‐13 gene define novel family of C2‐domain proteins. Journal of Biological Chemistry, 270(42), 25273–80. 10.1074/jbc.270.42.25273 7559667

[dneu22892-bib-0015] Butt, A. M. , Fern, R. F. , & Matute, C. (2014). Neurotransmitter signaling in white matter. Glia, 62(11), 1762–79. 10.1002/glia.22674 24753049

[dneu22892-bib-0016] Calloway, N. , Gouzer, G. , Xue, M. , & Ryan, T. A. (2015). The active‐zone protein Munc13 controls the use‐dependence of presynaptic voltage‐gated calcium channels. ELife, 4, (JULY2015), 10.7554/eLife.07728 PMC4525472 26196145

[dneu22892-bib-0017] Chen, Z. , Cooper, B. , Kalla, S. , Varoqueaux, F. , & Young, S. M. (2013). The munc13 proteins differentially regulate readily releasable pool dynamics and calcium‐dependent recovery at a central synapse. Journal of Neuroscience, 33(19), 8336–51. 10.1523/JNEUROSCI.5128-12.2013 23658173 PMC6619620

[dneu22892-bib-0018] Conner, J. M. , Lauterborn, J. C. , Yan, Q. , Gall, C. M. , & Varon, S. (1997). Distribution of brain‐derived neurotrophic factor (BDNF) protein and mRNA in the normal adult rat CNS: Evidence for anterograde axonal transport. Journal of Neuroscience, 17(7), 2295–2313. 10.1523/JNEUROSCI.17-07-02295.1997 9065491 PMC6573520

[dneu22892-bib-0019] Cooper, B. , Hemmerlein, M. , Ammermüller, J. , Imig, C. , Reim, K. , Lipstein, N. , Kalla, S. , Kawabe, H. , Brose, N. , Brandstätter, J. H. , & Varoqueaux, F. (2012). Munc13‐independent vesicle priming at mouse photoreceptor ribbon synapses. Journal of Neuroscience, 32(23), 8040–52. 10.1523/JNEUROSCI.4240-11.2012 22674279 PMC6620942

[dneu22892-bib-0020] Dagani, F. , & D'Angelo, E. (1992). Glutamate metabolism, release, and quantal transmission at central excitatory synapses: Implications for neural plasticity. Functional Neurology, 7(4), 315–336. 1358763

[dneu22892-bib-0021] Deken, S. L. , Beckman, M. L. , Boos, L. , & Quick, M. W. (2000). Transport rates of GABA transporters: Regulation by the N‐terminal domain and syntaxin 1A. Nature Neuroscience, 3(10), 998–1003. 10.1038/79939 11017172

[dneu22892-bib-0022] Delgado‐Martínez, I. , Nehring, R. B. , & Sørensen, J. B. (2007). Differential abilities of SNAP‐25 homologs to support neuronal function. Journal of Neuroscience, 27(35), 9380–9391. 10.1523/JNEUROSCI.5092-06.2007 17728451 PMC6673127

[dneu22892-bib-0023] Demarque, M. , Represa, A. , Becq, H. , Khalilov, I. , Ben‐Ari, Y. , & Aniksztejn, L. (2002). Paracrine intercellular communication by a Ca2+‐ and SNARE‐independent release of GABA and glutamate prior to synapse formation. Neuron, 36(6), 1051–1061. 10.1016/S0896-6273(02)01053-X 12495621

[dneu22892-bib-0024] Dhara, M. , Mohrmann, R. , & Bruns, D. (2018). v‐SNARE function in chromaffin cells. Pflügers Archiv ‐ European Journal of Physiology, 470(1), 169–180. 10.1007/s00424-017-2066-z 28887593 PMC5748422

[dneu22892-bib-0025] Dudok, J. J. , Groffen, A. J. A. , Toonen, R. F. T. , & Verhage, M. (2011). Deletion of Munc18‐1 in 5‐HT neurons results in rapid degeneration of the 5‐HT system and early postnatal lethality. Plos One, 6(11), e28137. 10.1371/journal.pone.0028137 22140524 PMC3226659

[dneu22892-bib-0026] Dulubova, I. , Sugita, S. , Hill, S. , Hosaka, M. , Fernandez, I. , Südhof, T. C. , & Rizo, J. (1999). A conformational switch in syntaxin during exocytosis: Role of munc18. The EMBO Journal, 18(16), 4372–4382. 10.1093/emboj/18.16.4372 10449403 PMC1171512

[dneu22892-bib-0027] Duman, J. G. , & Forte, J. G. (2003). What is the role of SNARE proteins in membrane fusion? Review Am J Physiol Cell Physiol, 285(2), C237–49. 10.1152/AJPCELL.00091.2003 12842832

[dneu22892-bib-0028] Dutta, D. J. , Woo, D. H. , Lee, P. R. , Pajevic, S. , Bukalo, O. , Huffman, W. C. , Wake, H. , Basser, P. J. , SheikhBahaei, S. , Lazarevic, V. , Smith, J. C. , & Fields, R. D. (2018). Regulation of myelin structure and conduction velocity by perinodal astrocytes. Proceedings of the National Academy of Sciences of the United States of America, 115(46), 11832–11837. 10.1073/pnas.1811013115. Epub 2018 Oct 29. 30373833 PMC6243273

[dneu22892-bib-0029] Engel, A. G. , Selcen, D. , Shen, X. M. , Milone, M. , & Harper, C. M. (2016). Loss of MUNC13‐1 function causes microcephaly, cortical hyperexcitability, and fatal myasthenia. Neurology: Genetics, 2(5), e105. 10.1212/NXG.0000000000000105 27648472 PMC5017540

[dneu22892-bib-0030] Faraone, S. V. , Perlis, R. H. , Doyle, A. E. , Smoller, J. W. , Goralnick, J. J. , Holmgren, M. A. , & Sklar, P. (2005). Molecular genetics of attention‐deficit/hyperactivity disorder. Biological Psychiatry, 57(11), 1313–1323. 10.1016/j.biopsych.2004.11.024 15950004

[dneu22892-bib-0031] Fatt, P. , & Katz, B. (1952). Spontaneous subthreshold activity at motor nerve endings. Journal of Physiology, 117(1), 109–128. 10.1113/jphysiol.1951.sp004675 14946732 PMC1392564

[dneu22892-bib-0032] Feldmann, A. , Amphornrat, J. , Schönherr, M. , Winterstein, C. , Möbius, W. , Ruhwedel, T. , Danglot, L. , Nave, K.‐A. , Galli, T. , Bruns, D. , Trotter, J. , & Krämer‐Albers, E.‐M. (2011). Transport of the major myelin proteolipid protein is directed by VAMP3 and VAMP7. The Journal of Neuroscience, 31(15), 5659LP–5672. 10.1523/JNEUROSCI.6638-10.2011 21490207 PMC6622839

[dneu22892-bib-0033] Fusco, C. , Micale, L. , Augello, B. , Pellico, M. T. , Menghini, D. , Alfieri, P. , Digilio, M. C. , Mandriani, B. , Carella, M. , Palumbo, O. , Vicari, S. , & Merla, G. (2013). Smaller and larger deletions of the Williams Beuren syndrome region implicate genes involved in mild facial phenotype, epilepsy and autistic traits. European Journal of Human Genetics 2014 22:1, 22(1), 64–70. 10.1038/ejhg.2013.101 PMC3865388 23756441

[dneu22892-bib-0034] Gaisano, H. Y. (2017). Recent new insights into the role of SNARE and associated proteins in insulin granule exocytosis. Diabetes, Obesity & Metabolism, 19, *Suppl 1*, 115–123. 10.1111/dom.13001 28880475

[dneu22892-bib-0035] Geerlings, A. , Pez‐Corcuera, B. L. , & Aragö, C. (2000). Characterization of the interactions between the glycine transporters GLYT1 and GLYT2 and the SNARE protein syntaxin 1A. FEBS Lett., 470(1), 51–54. 10.1016/s0014-5793(00)01297-7 10722844

[dneu22892-bib-0036] Gioia, D. A. , Alexander, N. J. , & McCool, B. A. (2016). Differential expression of munc13‐2 produces unique synaptic phenotypes in the basolateral amygdala of C57bl/6J and DBA/2J mice. Journal of Neuroscience, 36(43), 10964–10977. 10.1523/JNEUROSCI.1785-16.2016 27798178 PMC5098836

[dneu22892-bib-0037] Girod, R. , Popov, S. , Alder, J. , Zheng, J. Q. , Lohof, A. , & Poo, M. M. (1995). Spontaneous quantal transmitter secretion from myocytes and fibroblasts: Comparison with neuronal secretion. The Journal of Neuroscience : The Official Journal of the Society for Neuroscience, 15(4), 2826–2838. 10.1523/JNEUROSCI.15-04-02826.1995 7722632 PMC6577798

[dneu22892-bib-0038] Grant, E. , Hoerder‐Suabedissen, A. , & Molnar, Z. (2016). The regulation of corticofugal fiber targeting by retinal inputs. Cerebral Cortex, 26(3), 1336–1348. 10.1093/cercor/bhv315 26744542 PMC4737616

[dneu22892-bib-0039] Greenlee, M. H. W. , Roosevelt, C. B. , & Sakaguchi, D. S. (2001). Differential localization of SNARE complex proteins SNAP‐25, Syntaxin, and VAMP during development of the mammalian retina. Journal of Comparative Neurology, 10.1002/1096-9861(20010212)430:3<306::AID‐CNE1032>3.0.CO;2‐B 11169469

[dneu22892-bib-0040] Greenlee, M. H. W. , Wilson, M. C. , & Sakaguchi, D. S. (2002). Expression of SNAP‐25 during mammalian retinal development: Thinking outside the synapse. Seminars in Cell and Developmental Biology, 10.1016/S1084-9521(02)00015-0 12127142

[dneu22892-bib-0041] Groh, A. , de Kock, C. P. J. , Wimmer, V. C. , Sakmann, B. , & Kuner, T. (2008). Driver or coincidence detector: Modal switch of a corticothalamic giant synapse controlled by spontaneous activity and short‐term depression. Journal of Neuroscience, 28(39), 9652–9663. 10.1523/JNEUROSCI.1554-08.2008 18815251 PMC6671213

[dneu22892-bib-0042] Guiberson, N. G. L. , Pineda, A. , Abramov, D. , Kharel, P. , Carnazza, K. E. , Wragg, R. T. , Dittman, J. S. , & Burré, J. (2018). Mechanism‐based rescue of Munc18‐1 dysfunction in varied encephalopathies by chemical chaperones. Nature Communications, 10.1038/s41467-018-06507-4 PMC6162227 30266908

[dneu22892-bib-0043] Han, J. , Pluhackova, K. , & Böckmann, R. A. (2017). The multifaceted role of SNARE proteins in membrane fusion. Frontiers in Physiology, 10.3389/fphys.2017.00005 PMC5247469 28163686

[dneu22892-bib-0044] Han, J. , Yoon, S. , & Park, H. (2021). Endocytic BDNF secretion regulated by Vamp3 in astrocytes. Scientific Reports, 10.1038/s41598-021-00693-w PMC8551197 34707216

[dneu22892-bib-0045] Hanson, P. I. , Roth, R. , Morisaki, H. , Jahn, R. , & Heuser, J. E. (1997). Structure and conformational changes in NSF and its membrane receptor complexes visualized by quick‐freeze/deep‐etch electron microscopy. Cell, 90(3), 523–535. 10.1016/S0092-8674(00)80512-7 9267032

[dneu22892-bib-0046] Harris, W. A. (1984). Axonal pathfinding in the absence of normal pathways and impulse activity. The Journal of Neuroscience, 4(4), 1153LP–1162. 10.1523/JNEUROSCI.04-04-01153.1984 6325605 PMC6564780

[dneu22892-bib-0047] Hay, J. C. (2001). SNARE complex structure and function. Experimental Cell Research, 271(1), 10–21. 10.1006/excr.2001.5368 11697877

[dneu22892-bib-0048] Hayashi, S. , Hoerder‐Suabedissen, A. , Kiyokage, E. , Maclachlan, C. , Toida, K. , Knott, G. , & Molnár, Z. (2021). Maturation of complex synaptic connections of layer 5 cortical axons in the posterior thalamic nucleus requires SNAP25. Cerebral Cortex, 31(5), 2625–2638. 10.1093/cercor/bhaa379 33367517 PMC8023812

[dneu22892-bib-0049] He, E. , Wierda, K. , Van Westen, R. , Broeke, J. H. , Toonen, R. F. , Cornelisse, L. N. , & Verhage, M. (2017). Munc13‐1 and Munc18‐1 together prevent NSF‐dependent de‐priming of synaptic vesicles. Nature Communications, 8, 10.1038/ncomms15915 PMC5482055 28635948

[dneu22892-bib-0050] Hepp, R. , Perraut, M. , Chasserot‐Golaz, S. , Galli, T. , Aunis, D. , Langley, K. , & Grant, N. J. (1999). Cultured glial cells express the SNAP‐25 analogue SNAP‐23. Glia, 27(2), 181–187. 10.1002/(SICI)1098-1136(199908)27:2<181::AID‐GLIA8>3.0.CO;2‐9 10417817

[dneu22892-bib-0051] Herwegen, J. V. (2015). Williams syndrome and its cognitive profile: The importance of eye movements. Psychology Research and Behavior Management, 8, 143. 10.2147/PRBM.S63474 26082669 PMC4461016

[dneu22892-bib-0052] Hess, E. J. , Collins, K. A. , & Wilson, M. C. (1996). Mouse model of hyperkinesis implicates SNAP‐25 in behavioral regulation. Journal of Neuroscience, 16(9), 3104–3111. 10.1523/jneurosci.16-09-03104.1996 8622140 PMC6579059

[dneu22892-bib-0053] Hess, E. J. , Jinnah, H. A. , Kozak, C. A. , & Wilson, M. C. (1992). Spontaneous locomotor hyperactivity in a mouse mutant with a deletion including the Snap gene on chromosome 2. Journal of Neuroscience, 12(7), 2865–2874. 10.1523/jneurosci.12-07-02865.1992 1613559 PMC6575838

[dneu22892-bib-0054] Hines, J. H. , Ravanelli, A. M. , Schwindt, R. , Scott, E. K. , & Appel, B. (2015). Neuronal activity biases axon selection for myelination in vivo. Nature Neuroscience, 18, 683–689. 10.1038/nn.3992 25849987 PMC4414883

[dneu22892-bib-0055] Hoerder‐Suabedissen, A. , Korrell, K. V. , Hayashi, S. , Jeans, A. , Ramirez, D. M. O. , Grant, E. , Christian, H. C. , Kavalali, E. T. , Wilson, M. C. , & Molnár, Z. (2019). Cell‐specific loss of SNAP25 from cortical projection neurons allows normal development but causes subsequent neurodegeneration. Cerebral Cortex (New York, N.Y. : 1991), 29(5), 2148–2159. 10.1093/cercor/bhy127 PMC6754517 29850799

[dneu22892-bib-0056] Hoogland, P. V. , Wouterlood, F. G. , Welker, E. , & Van der Loos, H. (1991). Ultrastructure of giant and small thalamic terminals of cortical origin: A study of the projections from the barrel cortex in mice using Phaseolus vulgaris leuco‐agglutinin (PHA‐L). Experimental Brain Research, 87(1), 159–172. 10.1007/BF00228517 1721878

[dneu22892-bib-0057] Hughes, A. N. , & Appel, B. (2020). Microglia phagocytose myelin sheaths to modify developmental myelination. Nature Neuroscience, 23(9), 1055–1066. 10.1038/s41593-020-0654-2 32632287 PMC7483351

[dneu22892-bib-0058] Hume, R. I. , Role, L. W. , & Fischbach, G. D. (1983). Acetylcholine release from growth cones detected with patches of acetylcholine receptor‐rich membranes. Nature, 305(5935), 632–4. 10.1038/305632a0 6621712

[dneu22892-bib-0059] Imura, Y. , Morizawa, Y. , Komatsu, R. , Shibata, K. , Shinozaki, Y. , Kasai, H. , Moriishi, K. , Moriyama, Y. , & Koizumi, S. (2013). Microglia release ATP by exocytosis. Glia, 61(8), 1320–1330. 10.1002/glia.22517 23832620

[dneu22892-bib-0060] Irfan, M. , Evonuk, K. S. , & DeSilva, T. M. (2021). Microglia phagocytose oligodendrocyte progenitor cells and synapses during early postnatal development: Implications for white versus gray matter maturation. The FEBS Journal, 289(8), 2110–2127. 10.1111/febs.16190 34496137

[dneu22892-bib-0061] Ishii, A. , Furusho, M. , Macklin, W. , & Bansal, R. (2019). Independent and cooperative roles of the Mek/ERK1/2‐MAPK and PI3K/Akt/mTOR pathways during developmental myelination and in adulthood. Glia, 67(7), 1277–1295. 10.1002/glia.23602 30761608 PMC6571146

[dneu22892-bib-0062] Jeans, A. F. , Oliver, P. L. , Johnson, R. , Capogna, M. , Vikman, J. , Molnár, Z. , Babbs, A. , Partridge, C. J. , Salehi, A. , Bengtsson, M. , Eliasson, L. , Rorsman, P. , & Davies, K. E. (2007). A dominant mutation in Snap25 causes impaired vesicle trafficking, sensorimotor gating, and ataxia in the blind‐drunk mouse. Proceedings of the National Academy of Sciences of the United States of America, 104(7), 2431–2436. 10.1073/pnas.0610222104 17283335 PMC1793901

[dneu22892-bib-0063] Jena, B. P. (2011). Role of SNAREs in membrane fusion. Advances in Experimental Medicine and Biology, 10.1007/978-94-007-0763-4_3 21432012

[dneu22892-bib-0064] Katz, B. , & Miledi, R. (1963). A study of spontaneous miniature potentials in spinal motoneurones. The Journal of Physiology, 168(2), 389–422. 10.1113/jphysiol.1963.sp007199 14062684 PMC1359431

[dneu22892-bib-0065] Katz, L. C. , & Shatz, C. J. (1996). Synaptic activity and the construction of cortical circuits. Science (New York, N.Y.), 274(5290), 1133–1138. 10.1126/science.274.5290.1133 8895456

[dneu22892-bib-0066] Katz, L. C. , & Shatz, C. J. (1996). Synaptic activity and the construction of cortical circuits. Science, 274(5290), 1133–8. 10.1126/science.274.5290.1133 8895456

[dneu22892-bib-0067] Koch, H. , Hofmann, K. , & Brose, N. (2000). Definition of Munc13‐homology‐domains and characterization of a novel ubiquitously expressed Munc13 isoform. Biochemical Journal, 349(1), 247–253. 10.1042/0264-6021:3490247 10861235 PMC1221144

[dneu22892-bib-0068] Kossel, A. H. , Williams, C. V. , Schweizer, M. , & Kater, S. B. (1997). Afferent innervation influences the development of dendritic branches and spines via both activity‐dependent and non‐activity‐dependent mechanisms. Journal of Neuroscience, 17(16), 6314–24. 10.1523/JNEUROSCI.17-16-06314.1997 9236241 PMC6568345

[dneu22892-bib-0069] Korrell, K. V. , Disser, J. , Parley, K. , Vadisiute, A. , Requena‐Komuro, M. C. , Fodder, H. , Pollart, C. , Knott, G. , Molnár, Z. , & Hoerder‐Suabedissen, A. (2019). Differential effect on myelination through abolition of activity‐dependent synaptic vesicle release or reduction of overall electrical activity of selected cortical projections in the mouse. Journal of Anatomy, 235(3), 452–467. 10.1111/joa.12974 30901089 PMC6704270

[dneu22892-bib-0070] Kovacevic, J. , Maroteaux, G. , Schut, D. , Loos, M. , Dubey, M. , Pitsch, J. , Remmelink, E. , Koopmans, B. , Crowley, J. , Cornelisse, L. N. , Sullivan, P. F. , Schoch, S. , Toonen, R. F. , Stiedl, O. , & Verhage, M. (2018). Protein instability, haploinsufficiency, and cortical hyper‐excitability underlie STXBP1 encephalopathy. Brain, 141(5), 1350–1374. 10.1093/brain/awy046 29538625 PMC5917748

[dneu22892-bib-0071] Lang, T. , & Jahn, R. (2008). Core proteins of the secretory machinery. Handbook of Experimental Pharmacology, 184(184), 107–127. 10.1007/978-3-540-74805-2_5 18064413

[dneu22892-bib-0072] LaMantia, A. S. , & Rakic, P. (1990). Axon overproduction and elimination in the corpus callosum of the developing rhesus monkey. The Journal of Neuroscience: The Official Journal of the Society for Neuroscience, 10(7), 2156–2175. 10.1523/JNEUROSCI.10-07-02156.1990 2376772 PMC6570389

[dneu22892-bib-0073] Ma, C. , Li, W. , Xu, Y. , & Rizo, J. (2011). Munc13 mediates the transition from the closed syntaxin‐Munc18 complex to the SNARE complex. Nature Structural and Molecular Biology, 18(7), 542–549. 10.1038/nsmb.2047 PMC3087822 21499244

[dneu22892-bib-0074] Maffei, L. (2002). Plasticity in the visual system: Role of neurotrophins and electrical activity. Archives Italiennes de Biologie, 140(4), 341–6. PMID: 12228987. 12228987

[dneu22892-bib-0075] McMullan, S. M. , Phanavanh, B. , Li, G. G. , & Barger, S. W. (2012). Metabotropic glutamate receptors inhibit microglial glutamate release. ASN Neuro, 4(5), 10.1042/AN20120044 PMC3413012 22770428

[dneu22892-bib-0076] Matthews, G. (1996). Synaptic exocytosis and endocytosis: Capacitance measurements. Current Opinion in Neurobiology, 6(3), 358–364. 10.1016/S0959-4388(96)80120-6 8794078

[dneu22892-bib-0077] Mayer, A. , Wickner, W. , & Haas, A. (1996). Sec18p (NSF)‐driven release of Sec17p (α‐SNAP) can precede docking and fusion of yeast vacuoles. Cell, 85(1), 83–94. 10.1016/S0092-8674(00)81084-3 8620540

[dneu22892-bib-0078] McKinney, R. A. , Capogna, M. , Dürr, R. , Gähwiler, B. H. , & Thompson, S. M. (1999). Miniature synaptic events maintain dendritic spines via AMPA receptor activation. Nature Neuroscience, 2(1), 44–9. 10.1038/4548 10195179

[dneu22892-bib-0079] Micu, I. , Jiang, Q. , Coderre, E. , Ridsdale, A. , Zhang, L. , Woulfe, J. , Yin, X. , Trapp, B. D. , McRory, J. E. , Rehak, R. , Zamponi, G. W. , Wang, W. , & Stys, P. K. (2006). NMDA receptors mediate calcium accumulation in myelin during chemical ischaemia. Nature, 439, 988–992. 10.1038/nature04474 16372019

[dneu22892-bib-0080] Micu, I. , Ridsdale, A. , Zhang, L. , Woulfe, J. , McClintock, J. , Brantner, C. A. , Andrews, S. B. , & Stys, P. K. (2007). Real‐time measurement of free Ca2+ changes in CNS myelin by two‐photon microscopy. Nature Medicine, 13, 874–879. 10.1038/nm1568 17603496

[dneu22892-bib-0081] Mielnicka, A. , & Michaluk, P. (2021). Exocytosis in Astrocytes. Biomolecules, 11(9), 1367–1386. 10.3390/biom11091367 34572580 PMC8471187

[dneu22892-bib-0082] Mishima, T. , Fujiwara, T. , Sanada, M. , Kofuji, T. , Kanai‐Azuma, M. , & Akagawa, K. (2014). Syntaxin 1B, but Not Syntaxin 1A, Is Necessary for the Regulation of Synaptic Vesicle Exocytosis and of the Readily Releasable Pool at Central Synapses. Plos One, 9(2), 10.1371/JOURNAL.PONE.0090004 PMC3938564 24587181

[dneu22892-bib-0083] Miyamoto, N. , Maki, T. , Shindo, A. , Liang, A. C. , Maeda, M. , Egawa, N. , Itoh, K. , Lo, E. K. , Lok, J. , Ihara, M. , & Arai, K. (2015). Astrocytes promote oligodendrogenesis after white matter damage via brain‐derived neurotrophic factor. Journal of Neuroscience, 35(41), 14002–14008. 10.1523/JNEUROSCI.1592-15.2015 26468200 PMC4604233

[dneu22892-bib-0084] Miyamoto, A. , Wake, H. , Ishikawa, A. W. , Eto, K. , Shibata, K. , Murakoshi, H. , Koizumi, S. , Moorhouse, A. J. , Yoshimura, Y. , & Nabekura, J. (2016). Microglia contact induces synapse formation in developing somatosensory cortex. Nature Communications, 7, 10.1038/ncomms12540 PMC5007295 27558646

[dneu22892-bib-0085] Molnár, Z. , & Blakemore, C. (1991). Lack of regional specificity for connections formed between thalamus and cortex in coculture. Nature, 351(6326), 475–477. 10.1038/351475a0 2046749

[dneu22892-bib-0086] Molnár, Z. , & Blakemore, C. (1999). Development of signals influencing the growth and termination of thalamocortical axons in organotypic culture. Experimental Neurology, 156(2), 363–393. 10.1006/exnr.1999.7032 10328943

[dneu22892-bib-0087] Mozhayeva, M. G. , Sara, Y. , Liu, X. , & Kavalali, E. T. (2002). Development of vesicle pools during maturation of hippocampal synapses. Journal of Neuroscience, 22(3), 654–65. 10.1523/jneurosci.22-03-00654.2002 11826095 PMC6758530

[dneu22892-bib-0088] Netrakanti, P. R. , Cooper, B. H. , Dere, E. , Poggi, G. , Winkler, D. , Brose, N. , & Ehrenreich, H. (2015). Fast cerebellar reflex circuitry requires synaptic vesicle priming by Munc13‐3. Cerebellum (London, England), 14, 264–283. 10.1007/s12311-015-0645-0 PMC4441738 25617111

[dneu22892-bib-0089] Nicholson, K. L. , Munson, M. , Miller, R. B. , Filip, T. J. , Fairman, R. , & Hughson, F. M. (1998). Regulation of SNARE complex assembly by an N‐terminal domain of the t‐ SNARE Sso1p. Nature Structural Biology, 5, 264–283. 10.1038/1834 9731774

[dneu22892-bib-0090] Niemeyer, B. A. , & Schwarz, T. L. (2000). SNAP‐24, a Drosophila SNAP‐25 homologue on granule membranes, is a putative mediator of secretion and granule‐granule fusion in salivary glands. Journal of Cell Science, 113(22), 4055–4064. 10.1242/jcs.113.22.4055 11058092

[dneu22892-bib-0091] Noda, M. , Nakanishi, H. , Nabekura, J. , & Akaike, N. (2000). AMPA‐kainate subtypes of glutamate receptor in rat cerebral microglia. The Journal of Neuroscience: The Official Journal of the Society for Neuroscience, 20(1), 251–258. 10.1523/JNEUROSCI.20-01-00251.2000 10627602 PMC6774119

[dneu22892-bib-0092] Onodera, J. , Nagata, H. , Nakashima, A. , Ikegaya, Y. , & Koyama, R. (2021). Neuronal brain‐derived neurotrophic factor manipulates microglial dynamics. Glia, 69(4), 890–904. 10.1002/glia.23934 33119934

[dneu22892-bib-0093] Piper, M. , Plachez, C. , Zalucki, O. , Fothergill, T. , Goudreau, G. , Erzurumlu, R. , Gu, C. , & Richards, L. J. (2009). Neuropilin 1‐Sema signaling regulates crossing of cingulate pioneering axons during development of the corpus callosum. Cerebral Cortex (New York, NY), 19, (Suppl 1), i11. 10.1093/CERCOR/BHP027 PMC2693530 19357391

[dneu22892-bib-0094] Pletnikov, M. V. , Ayhan, Y. , Nikolskaia, O. , Xu, Y. , Ovanesov, M. V. , Huang, H. , Mori, S. , Moran, T. H. , & Ross, C. A. (2008). Inducible expression of mutant human DISC1 in mice is associated with brain and behavioral abnormalities reminiscent of schizophrenia. Molecular Psychiatry, 13(2), 173–186. 10.1038/sj.mp.4002079 17848917

[dneu22892-bib-0095] Pobbati, A. V. , Stein, A. , & Fasshauer, D. (2006). N‐ to C‐terminal SNARE complex assembly promotes rapid membrane fusion. Science, 10.1126/science.1129486 16888141

[dneu22892-bib-0096] Poirier, M. A. , Xiao, W. , Macosko, J. C. , Chan, C. , Shin, Y.‐K. , & Bennett, M. K. (1998). The synaptic SNARE complex is a parallel four‐stranded helical bundle. Nature Structural Biology, 5(9), 765–769. 9731768 10.1038/1799

[dneu22892-bib-0097] Quade, B. , Camacho, M. , Zhao, X. , Orlando, M. , Trimbuch, T. , Xu, J. , Li, W. , Nicastro, D. , Rosenmund, C. , & Rizo, J. (2019). Membrane bridging by munc13‐1 is crucial for neurotransmitter release. ELife, 313(5787), 673–676. 10.7554/eLife.42806 PMC6407922 30816091

[dneu22892-bib-0098] Quick, M. W. (2002). Role of syntaxin 1A on serotonin transporter expression in developing thalamocortical neurons; Role of syntaxin 1A on serotonin transporter expression in developing thalamocortical neurons. International Journal of Developmental Neuroscience, 20, 219–224. 10.1016/S0736-5748(02)00021-7 12175857

[dneu22892-bib-0099] Ramos‐Miguel, A. , Hercher, C. , Beasley, C. L. , Barr, A. M. , Bayer, T. A. , Falkai, P. , Leurgans, S. E. , Schneider, J. A. , Bennett, D. A. , & Honer, W. G. (2015). Loss of Munc18‐1 long splice variant in GABAergic terminals is associated with cognitive decline and increased risk of dementia in a community sample. Molecular Neurodegeneration, 10, 10.1186/s13024-015-0061-4 PMC4667524 26628003

[dneu22892-bib-0100] Raptis, A. , Torrejón‐Escribano, B. , Gómez De Aranda, I. , & Blasi, J. (2005). Distribution of synaptobrevin/VAMP 1 and 2 in rat brain. Journal of Chemical Neuroanatomy, 30(4), 201–211. 10.1016/j.jchemneu.2005.08.002 16169186

[dneu22892-bib-0101] Rizo, J. , & Südhof, T. C. (2012). The membrane fusion enigma: SNAREs, Sec1/Munc18 proteins, and their accomplices guilty as charged? Annual Review of Cell and Developmental Biology, 28, 279–308. 10.1146/annurev-cellbio-101011-155818 23057743

[dneu22892-bib-0102] Rojewska, E. , Piotrowska, A. , Popiolek‐Barczyk, K. , & Mika, J. (2018). Botulinum toxin type A—a modulator of spinal neuron–glia interactions under neuropathic pain conditions. Toxins, 10(4), 145. 10.3390/toxins10040145 PMC5923311 29614835

[dneu22892-bib-0103] Rossner, S. , Fuchsbrunner, K. , Lange‐Dohna, C. , Hartlage‐Rübsamen, M. , Bigl, V. , Betz, A. , Reim, K. , & Brose, N. (2004). Munc13‐1‐mediated vesicle priming contributes to secretory amyloid precursor protein processing. Journal of Biological Chemistry, 279(27), P27841–P27844. 10.1074/jbc.C400122200 15123597

[dneu22892-bib-0104] Sando, R. , Bushong, E. , Zhu, Y. , Huang, M. , Considine, C. , Phan, S. , Ju, S. , Uytiepo, M. , Ellisman, M. , & Maximov, A. (2017). Assembly of excitatory synapses in the absence of glutamatergic neurotransmission. Neuron, 94(2), 312–321.e3. 10.1016/j.neuron.2017.03.047 28426966 PMC5521186

[dneu22892-bib-0105] Santos, T. C. , Wierda, K. , Broeke, J. H. , Toonen, R. F. , & Verhage, M. (2017). Early golgi abnormalities and neurodegeneration upon loss of presynaptic proteins munc18‐1, syntaxin‐1, or SNAP‐25. Journal of Neuroscience, 37(17), 4525–4539. 10.1523/JNEUROSCI.3352-16.2017 28348137 PMC6596660

[dneu22892-bib-0106] Schoch, S. , Deák, F. , Königstorfer, A. , Mozhayeva, M. , Sara, Y. , Südhof, T. C. , & Kavalali, E. T. (2001). SNARE function analyzed in synaptobrevin/VAMP knockout mice. Science, 294(5544), 1117–1122. 10.1126/SCIENCE.1064335/SUPPL_FILE/1064335S2_THUMB.GIF 11691998

[dneu22892-bib-0107] Schubert, V. , Bouvier, D. , & Volterra, A. (2011). SNARE protein expression in synaptic terminals and astrocytes in the adult hippocampus: A comparative analysis. Glia, 59(10), 1472–88. 10.1002/glia.21190. Epub 2011 Jun 8.PMID: 21656854 21656854

[dneu22892-bib-0108] Schwarz, Y. , Zhao, N. , Kirchhoff, F. , & Bruns, D. (2017). Astrocytes control synaptic strength by two distinct v‐SNARE‐dependent release pathways. Nature Neuroscience, 20, 1529–1539. 10.1038/nn.4647. Epub 2017 Sep 25. 28945220

[dneu22892-bib-0109] Seeman, P. , Bzowej, N. H. , Guan, H. C. , Bergeron, C. , Becker, L. E. , Reynolds, G. P. , Bird, E. D. , Riederer, P. , Jellinger, K. , & Watanabe, S. (1987). Human brain dopamine receptors in children and aging adults. Synapse (New York, N.Y.), 1(5), 399–404. 10.1002/syn.890010503 3505371

[dneu22892-bib-0110] Sigler, A. , Oh, W. C. , Imig, C. , Altas, B. , Kawabe, H. , Cooper, B. H. , Kwon, H. B. , Rhee, J. S. , & Brose, N. (2017). Formation and maintenance of functional spines in the absence of presynaptic glutamate release. Neuron, 94(2), 304–311.e4. 10.1016/j.neuron.2017.03.029 28426965 PMC5418202

[dneu22892-bib-0111] Soares, C. , Lee, K. F. H. , Nassrallah, W. , & Béïque, J.‐C. (2013). Differential subcellular targeting of glutamate receptor subtypes during homeostatic synaptic plasticity. Journal of Neuroscience, 33(33), 13547–59. 10.1523/JNEUROSCI.1873-13.2013 23946413 PMC6705149

[dneu22892-bib-0112] Sharma, M. , Burré, J. , & Südhof, T. C. (2012). Proteasome inhibition alleviates SNARE‐dependent neurodegeneration. Science Translational Medicine, 4(147), 1–10. 10.1126/scitranslmed.3004028 22896677

[dneu22892-bib-0113] Shimojo, M. , Courchet, J. , Pieraut, S. , Torabi‐Rander, N. , Sando 3rd, R. , Polleux, F. , & Maximov, A. (2015). SNAREs Controlling vesicular release of BDNF and development of callosal axons. Cell Reports, 11(7), 1054–1066. 10.1016/j.celrep.2015.04.032 25959820 PMC4439258

[dneu22892-bib-0114] Söllner, T. , Bennett, M. K. , Whiteheart, S. W. , Scheller, R. H. , & Rothman, J. E. (1993). A protein assembly‐disassembly pathway in vitro that may correspond to sequential steps of synaptic vesicle docking, activation, and fusion. Cell, 75(3), 409–418. https://eutils.ncbi.nlm.nih.gov/entrez/eutils/elink.fcgi?dbfrom=pubmed&id=8221884&retmode=ref&cmd=prlinks%0Afile:///Files/91/918F253E-D90A-4BB2-A150-F3C0C4CFF846.pdf 8221884 10.1016/0092-8674(93)90376-2

[dneu22892-bib-0115] Sollner, T. , Whiteheart, S. W. , Brunner, M. , Erdjument‐bromage, H. , Geromanos, S. , Tempst, P. , & Rothman, J. E. (1993). SNAP receptors implicated in vesicle targeting and fusion. Nature, 362(6418), 318–324. 8455717 10.1038/362318a0

[dneu22892-bib-0116] Sons, M. S. , Verhage, M. , & Plomp, J. J. (2003). Role of Munc18‐1 in synaptic plasticity at the myasthenic neuromuscular junction. Annals of the New York Academy of Sciences, 1512(1), 10.1196/annals.1254.052 14592907

[dneu22892-bib-0117] Sørensen, J. B. , Nagy, G. , Varoqueaux, F. , Nehring, R. B. , Brose, N. , Wilson, M. C. , & Neher, E. (2003). Differential control of the releasable vesicle pools by SNAP‐25 splice variants and SNAP‐23. Cell, 114(1), 75–86. 10.1016/S0092-8674(03)00477-X 12859899

[dneu22892-bib-0118] Stryker, M. P. , & Harris, W. A. (1986). Binocular impulse blockade prevents the formation of ocular dominance columns in cat visual cortex. The Journal of Neuroscience, 6(8), 2117LP–2133. 10.1523/JNEUROSCI.06-08-02117.1986 3746403 PMC6568765

[dneu22892-bib-0119] Südhof, T. C. (2012). The presynaptic active zone. Neuron, 114(1), 75–86. 10.1016/j.neuron.2012.06.012 PMC3743085 22794257

[dneu22892-bib-0120] Südhof, T. C. , & Rothman, J. E. (2009). Membrane fusion: Grappling with SNARE and SM proteins. Science, 75(1), P11–25. 10.1126/science.1161748 PMC3736821 19164740

[dneu22892-bib-0121] Sutton, R. B. , Fasshauer, D. , Jahn, R. , & Brunger, A. T. (1998). Crystal structure of a SNARE complex involved in synaptic exocytosis at 2.4 A resolution. Nature, 395, (September), 347–353. 9759724 10.1038/26412

[dneu22892-bib-0122] Tafoya, L. C. R. , William, C. W. , Yanagawa, Y. , Obata, K. , & Wilson, M. C. (2008). The role of the t‐SNARE SNAP‐25 in action potential‐dependent calcium signaling and expression in GABAergic and glutamatergic neurons. BMC Neuroscience, 9, 10.1186/1471-2202-9-105 PMC2600647 18959796

[dneu22892-bib-0123] Tang, F. , Xiao, D. , Chen, L. , Gao, H. , & Li, X. (2021). Role of Munc18‐1 in the biological functions and pathogenesis of neurological disorders (Review). Molecular Medicine Reports, 23(3), 10.3892/mmr.2021.11837 PMC7821349 33495808

[dneu22892-bib-0124] Taylor, J. , Docherty, M. , & Gordon‐Weeks, P. R. (1990). GABAergic growth cones: Release of endogenous gamma‐aminobutyric acid precedes the expression of synaptic vesicle antigens. Journal of Neurochemistry, 54(5), 1689–1699. 10.1111/j.1471-4159.1990.tb01223.x 2109046

[dneu22892-bib-0125] Teng, F. Y. H. , Wang, Y. , & Tang, B. L. (2001). The syntaxins. Genome Biology, 2(11), 10.1186/gb-2001-2-11-reviews3012 PMC138984 11737951

[dneu22892-bib-0126] Ulloa, F. , Cotrufo, T. , Ricolo, D. , Soriano, E. , & Araújo, S. J. (2018). SNARE complex in axonal guidance and neuroregeneration. Neural Regeneration Research, 13(3), 386–392. 10.4103/1673-5374.228710 29623913 PMC5900491

[dneu22892-bib-0127] Urigüen, L. , Gil‐Pisa, I. , Munarriz‐Cuezva, E. , Berrocoso, E. , Pascau, J. , Soto‐Montenegro, M. L. , Gutiérrez‐Adán, A. , Pintado, B. , Madrigal, J. L. M. , Castro, E. , Sánchez‐Blázquez, P. , Ortega, J. E. , Guerrero, M. J. , Ferrer‐Alcon, M. , García‐Sevilla, J. A. , Micó, J. A. , Desco, M. , Leza, J. C. , Pazos, Á. , & Meana, J. J. (2013). Behavioral, neurochemical and morphological changes induced by the overexpression of munc18‐1a in brain of mice: Relevance to schizophrenia. Translational Psychiatry, 3(1), e221–e221. 10.1038/tp.2012.149 23340504 PMC3566728

[dneu22892-bib-0128] Vardar, G. , Chang, S. , Arancillo, M. , Wu, Y.‐J. , Trimbuch, T. , & Rosenmund, C. (2016). Distinct functions of Syntaxin‐1 in neuronal maintenance, synaptic vesicle docking, and fusion in mouse neurons. Journal of Neuroscience, 36(30), 7911–7924. 10.1523/JNEUROSCI.1314-16.2016 27466336 PMC6601879

[dneu22892-bib-0129] Varoqueaux, F. , Sigler, A. , Rhee, J. S. , Brose, N. , Enk, C. , Reim, K. , & Rosenmund, C. (2002). Total arrest of spontaneous and evoked synaptic transmission but normal synaptogenesis in the absence of Munc13‐mediated vesicle priming. Proceedings of the National Academy of Sciences of the United States of America, 99(13), 9037–9042. 10.1073/pnas.122623799 12070347 PMC124419

[dneu22892-bib-0130] Varoqueaux, F. , Sons, M. S. , Plomp, J. J. , & Brose, N. (2005). Aberrant morphology and residual transmitter release at the Munc13‐deficient mouse neuromuscular synapse. Molecular and Cellular Biology, 25(14), 10.1128/mcb.25.14.5973-5984.2005 PMC1168806 15988013

[dneu22892-bib-0131] Verderio, C. , Coco, S. , Bacci, A. , Rossetto, O. , De Camilli, P. , Montecucco, C. , & Matteoli, M. (1999). Tetanus toxin blocks the exocytosis of synaptic vesicles clustered at synapses but not of synaptic vesicles in isolated axons. Journal of Neuroscience, 19(16), 6723–6732. 10.1523/jneurosci.19-16-06723.1999 10436029 PMC6782867

[dneu22892-bib-0132] Verderio, C. , Coco, S. , Pravettoni, E. , Bacci, A. , & Matteoli, M. (1999). Synaptogenesis in hippocampal cultures. Cellular and Molecular Life Sciences : CMLS, 55(11), 1448–1462. 10.1007/s000180050384 10518992 PMC11146941

[dneu22892-bib-0133] Verhage, M. , Maia, A. S. , Plomp, J. J. , Brussaard, A. B. , Heeroma, J. H. , Vermeer, H. , Toonen, R. F. , Hammer, R. E. , Berg, T. K. V. D. , Missler, M. , Geuze, H. J. , & Südhof, T. C. (2000). Synaptic assembly of the brain in the absence of neurotransmitter secretion. Science, 287(5454), 864–869. 10.1126/SCIENCE.287.5454.864 10657302

[dneu22892-bib-0134] Vilinsky, I. , Stewart, B. A. , Drummond, J. , Robinson, I. , & Deitcher, D. L. (2002). A drosophila SNAP‐25 null mutant reveals context‐dependent redundancy with SNAP‐24 in neurotransmission. Genetics, 162(1), 259–271. 10.1093/genetics/162.1.259 12242238 PMC1462260

[dneu22892-bib-0135] Washbourne, P. , Thompson, P. M. , Carta, M. , Costa, E. T. , Mathews, J. R. , Lopez‐Benditó, G. , Molnár, Z. , Becher, M. W. , Valenzuela, C. F. , Partridge, L. D. , & Wilson, M. C. (2002). Genetic ablation of the t‐SNARE SNAP‐25 distinguishes mechanisms of neuroexocytosis. Nature Neuroscience, 5, 19–26. 10.1038/nn783 11753414

[dneu22892-bib-0136] Wake, H. , Moorhouse, A. J. , Miyamoto, A. , & Nabekura, J. (2013). Microglia: Actively surveying and shaping neuronal circuit structure and function. Trends in Neurosciences, 36(4), P209–217. 10.1016/j.tins.2012.11.007 23260014

[dneu22892-bib-0137] West, A. E. , & Greenberg, M. E. (2011). Neuronal activity‐regulated gene transcription in synapse development and cognitive function. Cold Spring Harbor Perspectives in Biology, 3(6), a005744. 10.1101/cshperspect.a005744. PMID: 21555405 21555405 PMC3098681

[dneu22892-bib-0138] Wilhelm, A. , Volknandt, W. , Langer, D. , Nolte, C. , Kettenmann, H. , & Zimmermann, H. (2004). Localization of SNARE proteins and secretory organelle proteins in astrocytes in vitro and in situ. Neuroscience Research, 48(3), 249–57. 10.1016/j.neures.2003.11.002. PMID: 15154671 15154671

[dneu22892-bib-0139] Wong, A. W. , Xiao, J. , Kemper, D. , Kilpatrick, T. J. , & Murray, S. S. (2013). Oligodendroglial expression of TrkB independently regulates myelination and progenitor cell proliferation. Journal of Neuroscience, 33(11), 4947–4957. 10.1523/JNEUROSCI.3990-12.2013 23486965 PMC6619007

[dneu22892-bib-0140] Wong, F. K. , Bercsenyi, K. , Sreenivasan, V. , Portalés, A. , Fernández‐Otero, M. , & Marín, O. (2018). Pyramidal cell regulation of interneuron survival sculpts cortical networks. Nature, 557(7707), 668–673. 10.1038/s41586-018-0139-6 29849154 PMC6207348

[dneu22892-bib-0141] Xiao, J. , Kilpatrick, T. J. , & Murray, S. S. (2009). The role of neurotrophins in the regulation of myelin development. Neuro‐Signals, 17(4), 265–276. 10.1159/000231893 19816063

[dneu22892-bib-0142] Yoon, T. Y. , & Munson, M. (2018). SNARE complex assembly and disassembly. Current Biology, 28(8), R397–R401. 10.1016/J.CUB.2018.01.005 29689222

[dneu22892-bib-0143] Young, S. H. , & Poo, M. M. (1983). Spontaneous release of transmitter from growth cones of embryonic neurones. Nature, 305(5935), 634–7. 6312327 10.1038/305634a0

[dneu22892-bib-0144] Zikich, D. , Mezer, A. , Varoqueaux, F. , Sheinin, A. , Junge, H. J. , Nachliel, E. , Melamed, R. , Brose, N. , Gutman, M. , & Ashery, U. (2008). Vesicle priming and recruitment by ubMunc13‐2 are differentially regulated by calcium and calmodulin. Journal of Neuroscience, 28(8), 1949–1960. 10.1523/JNEUROSCI.5096-07.2008 18287511 PMC6671433

[dneu22892-bib-0145] Zylbersztejn, K. , Petkovic, M. , Burgo, A. , Deck, M. , Garel, S. , Marcos, S. , Bloch‐Gallego, E. , Nothias, F. , Serini, G. , Bagnard, D. , Binz, T. , & Galli, T. (2012). The vesicular SNARE Synaptobrevin is required for Semaphorin 3A axonal repulsion. Journal of Cell Biology, 196(1), 37–46. 10.1083/JCB.201106113/VIDEO-1 22213797 PMC3255983

